# Stress before conception and during pregnancy and maternal cortisol during pregnancy: A scoping review

**DOI:** 10.1016/j.psyneuen.2023.106115

**Published:** 2023-04-18

**Authors:** Gabrielle R. Rinne, Jenna Hartstein, Christine M. Guardino, Christine Dunkel Schetter

**Affiliations:** a Department of Psychology, University of California, Los Angeles, Los Angeles, CA, USA; b Department of Psychology, Dickinson College, Carlisle, PA, USA

**Keywords:** Prenatal stress, Early life stress, Preconception stress, Cortisol, HPA axis

## Abstract

**Background:**

Stress before conception and during pregnancy is associated with less favorable maternal and child health. Alterations in prenatal cortisol levels may serve as a central biological pathway linking stress to adverse maternal and child health. Research examining associations between maternal stress from childhood through pregnancy and prenatal cortisol has not been comprehensively reviewed.

**Method:**

The current scoping review of 48 papers synthesizes studies reporting on associations between stress before conception and during pregnancy with maternal cortisol in pregnancy. Eligible studies measured childhood, the proximal preconception period, pregnancy, or lifetime stress based on stress exposures or appraisals and measured cortisol in saliva or hair during pregnancy.

**Results:**

Higher maternal childhood stress was associated with higher cortisol awakening responses and alterations in typical pregnancy-specific changes in diurnal cortisol patterns across studies. In contrast, most studies of preconception and prenatal stress reported null associations with cortisol and those reporting significant effects were inconsistent in direction. A few studies found that the associations between stress and cortisol during pregnancy varied as a function of several moderators including social support and environmental pollution.

**Conclusions:**

Although many studies have evaluated effects of maternal stress on prenatal cortisol, this scoping review is the first to synthesize existing literature on this topic. The association between stress before conception and during pregnancy and prenatal cortisol may depend on the developmental timing of stress and several moderators. Maternal childhood stress was more consistently associated with prenatal cortisol than proximal preconception or pregnancy stress. We discuss methodological and analytic factors that may contribute to mixed findings.

## 1. Introduction

Life course approaches propose that maternal health in pregnancy is shaped by interactions between risk and protective factors over the lifespan (Bernstein and Merkatz, 2010). Higher levels of maternal stress prior to conception, including both in the proximal preconception period (i.e., six to twelve months before pregnancy) as well as distal experiences in a mother’s own childhood, and during pregnancy predicts less favorable mental and physical health in pregnancy (Kane et al., 2018; Keenan et al., 2018; Racine et al., 2021; Rich-Edwards et al., 2006). The effects of maternal stress may also extend to offspring. Higher maternal stress before conception and during pregnancy increases the risk for low birth weight, preterm birth, and offspring mental and physical health problems (Davis and Narayan, 2020; Keenan et al., 2018; Staneva et al., 2015; van den Bergh et al., 2020). Dysregulation of the hypothalamic-pituitary-adrenal (HPA) axis is hypothesized to serve as a primary mechanism through which life course stress influences maternal-fetal health in the prenatal period and subsequent offspring development (Cottrell and Seckl, 2009; de Weerth and Buitelaar, 2005; Zijlmans et al., 2015). However, empirical investigations on maternal stress and cortisol levels in pregnancy have yet to be comprehensively reviewed. The current scoping review identifies and synthesizes existing literature on the associations of maternal stress from childhood through pregnancy with cortisol in pregnancy.

Cortisol is the hormonal end product of the HPA axis, a coordinated stress response system consisting of the hypothalamus, pituitary gland, and adrenal gland. HPA axis activation mobilizes physiological, psychological, and behavioral responses to stressors in the context of threat or challenges. The HPA axis also supports the functioning of various physiological systems in non-stress conditions, including the metabolic and immune systems (Demorrow, 2018). The multiple functions of the HPA axis can be indexed with different measures of cortisol: phasic, tonic, and diurnal. Phasic cortisol reflects responses to acute stressors whereas tonic or basal cortisol levels provide a measure of cortisol in neutral conditions. Tonic cortisol levels can be measured through single timepoint measures of salivary cortisol levels, total cortisol output over the course of the day, or through hair cortisol concentrations (Short et al., 2016). Tonic cortisol levels based on saliva samples collected at a single timepoint can vary substantially within individuals from day-to-day due to a number of factors including food intake and time of day (Dahlgren et al., 2009; Hruschka et al., 2005). Whereas salivary cortisol levels reflect cortisol output over a short period of time, hair cortisol concentrations reflect a measure of cortisol output over period of up to several months (Short et al., 2016). Tonic cortisol is regulated by central circadian control that contributes to variations in salivary cortisol levels over the course of the day (diurnal rhythm). The diurnal rhythm can be measured through repeated measurements of cortisol over the course of the day and is characterized by high levels of cortisol at waking, a steep increase 30–45 min after waking (cortisol awakening response), and a decline from morning until bedtime (diurnal slope) (Adam et al., 2017; Wilhelm et al., 2007).

During pregnancy, the HPA axis undergoes substantial shifts, largely due to the development of the placenta, which serves as a temporary endocrine organ (Burton & Fowden, 2015; Sandman, 2015). The placenta expresses a gene for corticotropin-releasing hormone (CRH) production thereby resulting in changes in HPA axis regulation. Specifically, placental CRH production shifts regulation of the maternal HPA axis from a negative feedback loop that is characteristic in the non-pregnant state to a tissue-specific positive feedback loop (for a review, see Sandman et al., 2018). The release of cortisol from the adrenal glands stimulates release of placental CRH (pCRH), resulting in simultaneous increases in CRH, adrenocorticotropic hormone (ACTH), and cortisol over the course of pregnancy that reach peak levels near the time of labor and delivery (for a review, see Howland et al., 2017). Consequently, there are substantial changes in tonic, phasic, and diurnal cortisol from early to late pregnancy. For example, cortisol levels increase nearly three-fold across pregnancy before returning to pre-pregnancy levels within 5–7 days of delivery (for reviews, see Duthie and Reynolds, 2013; Glynn et al., 2018; Howland et al., 2017). These increases in tonic cortisol are mirrored by changes in HPA axis reactivity and diurnal cortisol patterns. There is a dampening of phasic cortisol responses to stress, an attenuation of the cortisol awakening response, and a flattening of the cortisol slope from early to late pregnancy (de Weerth and Buitelaar, 2005; Entringer et al., 2010; Howland et al., 2017).

HPA axis changes that occur during pregnancy, including increases in stress hormone levels, facilitate changes in the maternal brain in the perinatal period, fetal development, and childbirth (for reviews, see Glynn et al., 2018; Howland et al., 2017; Sandman, 2018). Individual dysregulation in cortisol levels and trajectories, however, can predict adverse maternal mental health and birth outcomes, including less favorable maternal perinatal mental health, lower birth weight, and shorter length of gestation (Bolten et al., 2011; D’Anna-Hernandez et al., 2012; Dickens and Pawluski, 2018; Gilles et al., 2018; Yim et al., 2015). Placental 11 β hydroxysteroid dehydrogenase Type 2 (11β-HSD-2), an enzyme that converts maternal cortisol into its inactive metabolite cortisone, is a key regulator of fetal glucocorticoid exposure (Togher et al., 2014). Although higher levels of 11β-HSD-2 can protect the fetus from exposure to maternal cortisol, placental 11β-HSD-2 only serves as a partial barrier and an estimated 15% of maternal cortisol crosses the placenta unmetabolized and reaches the fetus (Howland et al., 2017). Therefore, elevated maternal cortisol levels and dysregulation of diurnal patterns can lead to increased fetal exposure to glucocorticoids. Excessive fetal exposure to cortisol, particularly in the first and second trimester, is associated with more fearful infant temperament, altered infant biobehavioral reactivity as well as differences in neurodevelopment and more internalizing problems in childhood and adolescence (for reviews, see Zijlmans et al., 2015 and Howland et al., 2017). Identifying the factors that shape cortisol levels and patterns in pregnancy is critical given the implications for maternal-fetal health and offspring development.

The stress responsive nature of the HPA axis and evidence of links between dysregulated cortisol levels and maternal-fetal health has led researchers to propose that the activity of the HPA axis during pregnancy is a mechanism linking stress to adverse perinatal outcomes (Cottrell and Seckl, 2009; de Weerth and Buitelaar, 2005). Although this topic has been a focus of many empirical studies, equivocal findings on the associations of stress with cortisol levels in pregnancy have led some researchers to question the hypothesis that dysregulated cortisol levels during pregnancy are a biological mechanism linking maternal stress to maternal-fetal health outcomes (O’Donnell and Meaney, 2017; Peterson et al., 2020). However, one recent systematic review of 25 papers synthesized literature on associations of stress in childhood with cortisol levels in pregnancy and found that childhood stress was associated with a greater cortisol awakening response in four of the five studies reviewed and blunted phasic cortisol in three studies reviewed (Epstein et al., 2021). Consistent with the Adaptive Calibration Model (del Giudice et al., 2011), a theoretical framework that proposes that childhood experiences calibrate physiological responses to stress across the lifespan, this review also found that childhood stress modified HPA axis regulation in the context of later stress and psychological distress. For example, childhood stress was associated with higher tonic cortisol levels in the context of higher psychological distress (e.g., depressive symptoms, dissociative symptoms, PTSD symptoms), low socioeconomic status, and adulthood trauma (Epstein et al., 2021). This prior review provides evidence that stress in childhood predicts HPA axis regulation and cortisol levels in pregnancy, but additional review papers that examine stress across developmental periods beyond childhood are necessary to elucidate how lifespan stress relates to cortisol levels in pregnancy.

## 2. The current study

In the present scoping review, we identify and summarize studies examining associations of maternal stress from childhood through pregnancy with tonic cortisol levels and diurnal cortisol indices in pregnancy as measured in saliva and hair. We include studies that evaluate measures of stress from childhood, the proximal preconception period, and pregnancy to extend upon the findings of a recent systematic review by Epstein and colleagues (2021). Consistent with established theories of stress, we conceptualize stress as a process involving demands that are appraised to exceed resources. This process includes stressors that vary in duration from acute events or episodic stressors to ongoing conditions or chronic stressors, as well as individuals’ appraisals or perceptions of their ability to cope with these demands (Lazarus and Folkman, 1984). Therefore, in the current review we include studies that measure stress exposures occurring from childhood through pregnancy as well as preconception and prenatal appraisals or perceptions of stress. Our aim is to provide an overview of how stress across development relates to cortisol levels in pregnancy, identify research gaps, and provide recommendations for future research.

## 3. Methods

The present scoping review followed Preferred Reporting Items for Systematic Reviews and Meta-Analyses (PRISMA) extension guidelines for scoping reviews (Tricco et al., 2018). The association between maternal stress and cortisol levels in pregnancy has been the focus of many empirical investigations with varying methodologies. Therefore, a scoping review was selected as the most appropriate review method to determine the scope of the existing literature, identify key concepts and gaps in the existing literature, and offer directions for future research.

### 3.1. Eligibility criteria

We included studies that examined associations of hair or salivary cortisol during pregnancy with stress from childhood through pregnancy. Articles were eligible for inclusion if the following criteria were met: (a) included at least one measure of stress exposures (i.e., exposure to environmental stressors, events, or conditions) from childhood through pregnancy or of stress appraisals or perceptions; (b) included at least one measure of hair or salivary cortisol collected during pregnancy (or at delivery for hair samples); and (c) analyzed and reported on the statistical association of stress and cortisol. To allow for comparison across studies, we focused on cortisol indices measured in saliva or hair as these were the primary measures of cortisol in pregnancy. Tonic measures of cortisol included (1) single timepoint measures of salivary cortisol levels, (2) total cortisol output over the course of the day from multiple saliva samples, and (3) hair cortisol concentrations. Diurnal cortisol indices captured patterns of change in cortisol levels over the course of the day due to the circadian rhythm and included the cortisol awakening response and slope calculated from multiple saliva samples. Other measures of cortisol in the literature included plasma (*n* = 3), serum (*n* = 1), urinary (*n* = 1), and amniotic fluid cortisol (*n* = 1).

Review papers, qualitative papers, protocol papers, and papers reporting on associations in exclusively adolescent samples or non-human samples were not included. We also excluded studies that operationalized stress as psychological distress (e.g., depression, anxiety, general distress) or included stress measures in a composite with psychological distress (*n* = 8). Associations of psychological distress with cortisol in pregnancy are reviewed elsewhere (see Khoury et al., 2022; Mustonen et al., 2018; Seth et al., 2016). We excluded these studies for this reason and several other reasons. First, from a theoretical perspective, stress exposures and appraisals (perceptions) of stress are defined differently from affective states or mental health symptoms (Epel et al., 2018; Lazarus and Folkman, 1984). Second, stress and affective states may have distinct associations with prenatal stress physiology (O’Donnell and Meaney, 2017). Nonetheless, it is also important to note that perceived stress and mental health symptoms are often moderately correlated, which should be kept in mind when interpreting results of specific studies.

### 3.2. Search method

We searched three databases for eligible studies: PsycInfo, PubMed, and CINAHL. In addition, we examined reference lists of records identified in databases and searched Google Scholar for additional eligible studies. Search terms are presented in Table 1. We conducted the database search in January 2022 and included articles published from database conception through January 2022. Studies in the grey literature and those that were not published in English were not included in the search to increase feasibility of the search process, as is common for scoping reviews (Pham et al., 2014).

### 3.3. Study selection

**Fig. 1.**
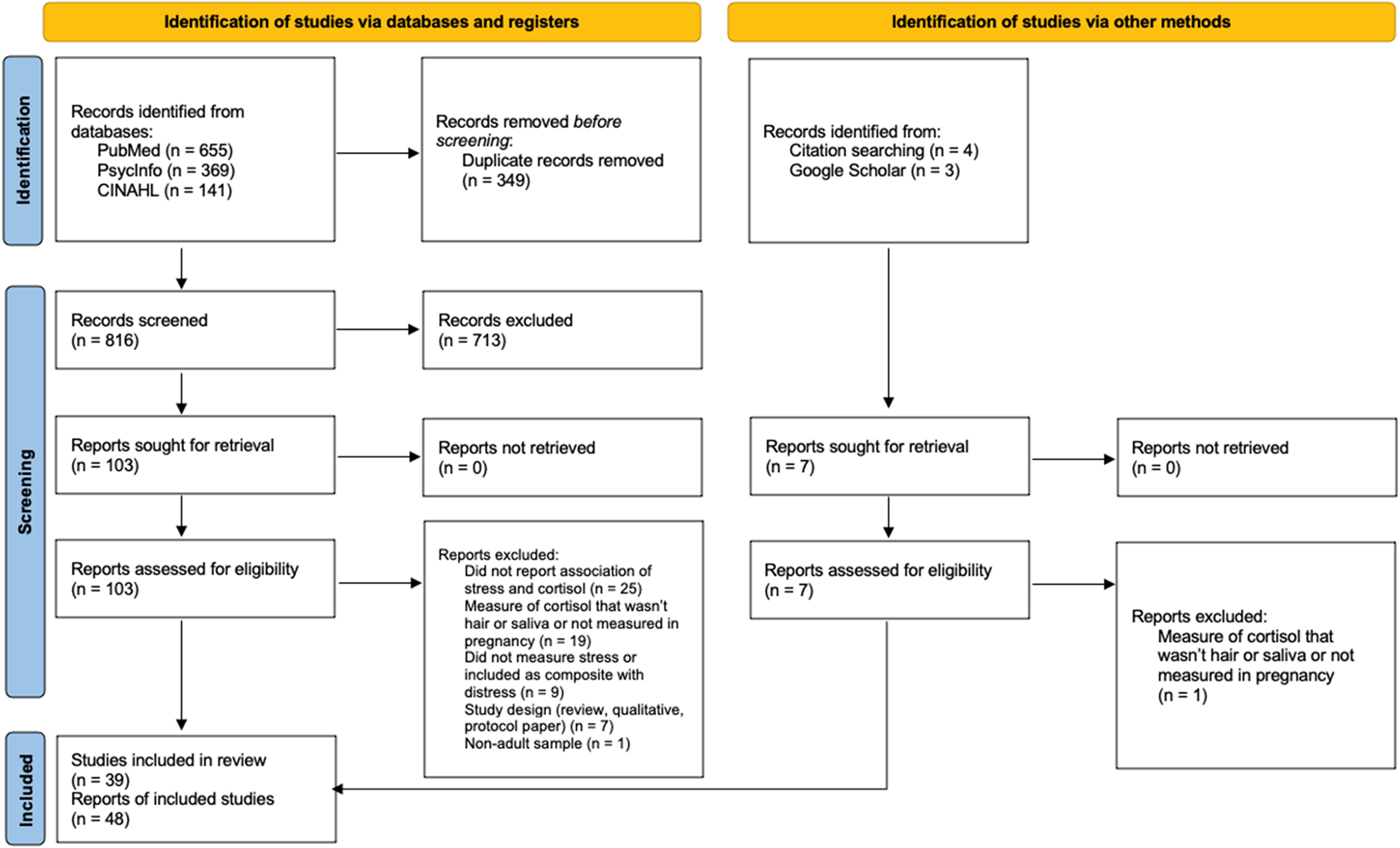
Flow diagram for the scoping review process.

In total, we identified 1165 records from databases, 816 of which were screened after 349 duplicates were removed. Two reviewers (one doctoral student in psychology with expertise in psychoneuroendocrinology in pregnancy, one senior-level undergraduate student in human biology) independently screened titles and abstracts for eligibility. If a reviewer was uncertain of eligibility for inclusion, reviewers met to resolve discrepancies and reach consensus. 713 records were excluded from title and abstract screening. 103 reports were sought for retrieval and assessed for eligibility with a full text review. Following full text review, 61 records were excluded, resulting in 42 studies that met final inclusion criteria from database search. Six studies were identified as eligible for inclusion from other search methods and therefore 48 studies were included in the scoping review. Fig. 1 presents detailed information about the search process and reasons for exclusion.

### 3.4. Data extraction

A data extraction form was developed, tested, and revised as necessary by two members of the research team. Once finalized, data was extracted from eligible studies by these team members. Variables extracted from each study included: (a) study setting; (b) sample sociodemographic characteristics (age, income and/or education, race/ethnicity, relationship status); (c) measure of stress and timing of assessment; (d) measure of cortisol and timing of assessment; (e) statistical analysis for associations of stress and cortisol; and (f) primary study results.

## 4. Results

### 4.1. Description of included studies

Forty-eight records, describing 39 unique cohorts, were identified as eligible for inclusion in the review. Articles were published between 2004 and 2022. Most studies were conducted in samples of individuals living in the United States (52.1%); however, there was global representation across studies (16.7% Germany; 12.5% Canada; 6.25% in Denmark, 12.4% other countries). Most reports were from prospective, longitudinal studies (*n* = 34), two studies were testing interventions, and the remainder were from cross-sectional studies (*n* = 12). Sample sizes ranged from pilot studies of 17 participants (Bublitz et al., 2016) to larger cohorts with 1587 participants (Harville et al., 2009). Participant mean age ranged from 21.6 years to 32.7 years.^1^ Most studies in this review involved samples of healthy, low-risk women. Six reports involved samples that were characterized as higher risk due to medical factors (Cesta et al., 2018; Swales et al., 2018), mental health problems or psychopathology (King et al., 2008; Nyström-Hansen et al., 2019; Shea et al., 2007), and/or sociodemographic characteristics (first-time mothers who were single and have low income; Bowers et al., 2018).

Thirty studies measured cortisol in saliva during pregnancy and 18 studies measured hair cortisol concentrations in pregnancy. Of those measuring salivary cortisol, 20 collected multiple measurements of cortisol over the course of the day and calculated diurnal indices (cortisol awakening response, slope) and 12 of the 20 also examined tonic cortisol (single timepoint levels or total cortisol output based on area under the curve from multiple measures over the day). That is, 12 studies examined associations of stress with the cortisol awakening response or slope (i.e., diurnal cortisol indices) as well as associations of stress with single measurements of cortisol or total cortisol output (i.e., tonic cortisol). None of the studies evaluating cortisol reactivity were eligible for inclusion in the present review, either because they included measures of cortisol that were not from hair or saliva (*n* = 2), did not report on the statistical association of stress with cortisol reactivity (*n* = 6), or operationalized stress as a composite with psychological distress (*n* = 1). Ten additional studies measured tonic cortisol only. Most studies included repeated assessments of cortisol at multiple time points in pregnancy (*n* = 31). Further detail on cortisol sample collection and primary results of studies included in the review are summarized in Table 2 with further detail provided in Table 3.

### 4.2. Synthesis of results

Measures of stress included measures of childhood stress, proximal preconception and pregnancy stress, and lifetime stress. We present results by developmental period, defined below. Most studies measured stress in within one year of pregnancy (hereafter, proximal preconception period) or pregnancy (*n* = 26), 11 studies measured stress in childhood and the proximal preconception period or pregnancy, 7 studies measured childhood stress only, and 4 studies measured lifetime stress. A subset of studies also evaluated moderators of the effects of stress on cortisol in pregnancy; interactive effects are presented separately, and main effects are presented in respective sections if they were not qualified by an interaction. In studies where multiple analyses were conducted, we present results of controlled analyses that adjust for covariates. Further detail on covariates is presented in Table 3.

### 4.3. Childhood stress

Studies assessing childhood stress included measures completed during pregnancy providing retrospective reports of stress occurring prior to the age of 18. Eighteen studies evaluated associations of childhood stress with cortisol in pregnancy. The Adverse Childhood Experiences Questionnaire (*n* = 7) and Childhood Trauma Questionnaire (*n* = 5) were most often used to assess childhood stress. These measures assess a range of childhood experiences, including parental divorce, parental mental illness, parental incarceration, and more severe forms of stress such as abuse and neglect.

#### Diurnal Cortisol.

Childhood stress was associated with a higher cortisol awakening response during pregnancy in five studies (Epstein et al., 2020; Stephens et al., 2021; Thomas et al., 2018a; 2018b; 2021). Childhood sexual abuse was significantly associated with a greater cortisol awakening response in early pregnancy (12–21 weeks; *n* = 178) (Stephens et al., 2021) and more stressors in childhood were also associated with a larger cortisol awakening response in mid pregnancy (M=26.5 weeks; *n* = 58) (Epstein et al., 2020). In both studies, results remained consistent when adjusting for recent stressors.

The other three studies reporting significant associations between childhood stress and the cortisol awakening response included participants enrolled in the Alberta Pregnancy Outcomes and Nutrition Study (APRON).^2^ Adverse childhood experiences were associated with a higher cortisol awakening response in early (M=15.1 weeks) and late pregnancy (M=32.5 weeks) in a sample of 248 individuals (Thomas-Argyriou et al., 2021). Similarly, in a larger sample of 356 individuals, adverse childhood experiences were associated with a higher cortisol awakening response in early pregnancy (M=11.4 weeks) (Thomas et al., 2018b). In another sample from this cohort (*n* = 243), adverse childhood experiences were associated with a higher cortisol awakening response in early pregnancy (M=15.1 weeks), but not late pregnancy (M=32.5 weeks; Thomas et al., 2018a). Thus, results in this cohort are largely consistent across reports in that childhood stress was associated with a larger cortisol awakening response across studies but indicate there may be differences by gestational timing. Differences across reports may also be attributable to differences in covariates included in each model or variation in the sample and sample size.

There was also some evidence that childhood stress predicts changes in diurnal cortisol indices across pregnancy. In a sample of 135 pregnant individuals, those with a history of childhood sexual abuse had larger increases in cortisol awakening responses from mid (M=24 weeks) to late pregnancy (M=35 weeks) (Bublitz and Stroud, 2012). Conversely, childhood stress was not associated with changes in the cortisol awakening response from early to late pregnancy (Thomas et al., 2018b), but was associated with less flattening of the diurnal slope from the first (M=11.4 weeks) to the third trimester (M=32.4 weeks) in a sample of 356 individuals (Thomas et al., 2018b). However, this association was no longer statistically significant when adjusting for prenatal stressful life events. In another study of 178 women, childhood economic disadvantage, but not childhood maltreatment, was associated with a flatter cortisol slope and less flattening of the cortisol slope between the second and third trimester (*n* = 178; Stephens et al., 2021). Together, these studies provide evidence that childhood stress may be associated with pregnancy-specific changes in the cortisol awakening response and diurnal slope such that childhood stress is associated with less flattening of the diurnal slope from early to late pregnancy and less attenuation of the cortisol awakening response from early to late pregnancy. Two studies reported null associations of adverse childhood experiences with cortisol slope (Jairaj et al., 2020; Thomas-Argyriou et al., 2021) and one study reported null associations of child abuse with changes in the cortisol slope from mid to late pregnancy (Bublitz and Stroud, 2012).

#### Tonic Cortisol: Salivary.

Associations of childhood stress with tonic levels of salivary cortisol were less consistent as compared to associations with diurnal indices. Regarding single measures of cortisol, childhood stress was associated with lower waking cortisol levels in mid to late pregnancy (M=28.4 weeks) (*n* = 66) (Shea et al., 2007) and higher levels of cortisol 30 min after waking (*n* = 356) (Thomas et al., 2018b). These findings on associations of childhood stress with lower waking cortisol levels and higher levels of cortisol 30 min after waking are consistent with associations of childhood stress with a greater prenatal cortisol awakening response (Bublitz and Stroud, 2012; Thomas-Argyriou et al., 2021; Thomas et al., 2018a; 2018b). Childhood stress was associated with higher evening cortisol levels in mid pregnancy (M=26 weeks) (*n* = 118) (Jairaj et al., 2020). Other studies reported null associations of childhood stress with cortisol levels at waking (Stephens et al., 2021), 30 min after waking (Bublitz and Stroud, 2012, 2013), and evening cortisol levels (Bublitz and Stroud, 2012; King et al., 2008).

Childhood stress was not significantly associated with total cortisol output in any of the studies testing this association (Thomas et al., 2018a; 2018b; Thomas-Argyriou et al., 2021).

#### Tonic Cortisol: Hair.

Three studies reported that childhood stress was associated with hair cortisol concentrations, but the direction of the effect differed across studies. In one study, individuals who experienced physical or sexual abuse in childhood had higher hair cortisol over the course of pregnancy compared to individuals who did not experience any form of childhood abuse (*n* = 180; hair cortisol collected just after delivery and segmented to reflect all three trimesters, the second and third trimester, or third trimester; Schreier et al., 2015a). However, childhood emotional abuse was not associated with hair cortisol concentrations. In stratified analyses by race/ethnicity, the association between childhood sexual or physical abuse and hair cortisol was statistically significant in Black participants but not White or Hispanic participants. Conversely, greater childhood stress was associated with marginally lower hair cortisol (hair samples collected at 18.7–38.9 weeks and segmented to reflect the prior four months of pregnancy) in a smaller sample of 44 individuals (Nyström-Hansen et al., 2019). Similarly, adverse childhood experiences were associated with lower hair cortisol based on hair samples collected in mid-pregnancy that were segmented to reflect the prior three months (*n* = 30) (Bowers et al., 2018). Two studies reported that childhood stress was not associated with hair cortisol (Schury et al., 2017 [collected within 6 days of delivery and segmented to reflect the third trimester]; Orta et al., 2019 [collected at time of delivery and segmented to reflect the first, second, and third trimester]).

### 4.4. Proximal preconception and pregnancy stress

Proximal preconception and pregnancy stress included assessments of stress in which participants reported on stress that occurred during pregnancy and/or during pregnancy and in the year before pregnancy. While all studies with measures of proximal preconception period stress included measures within a year of pregnancy, the range of proximal preconception period varied across studies. For example, some studies asked participants to report on stress in the past year in mid pregnancy (Stephens et al., 2021) whereas others asked participants to report on stress in the past six months in mid pregnancy (Shea et al., 2007). Thirty-seven studies evaluated associations of proximal preconception or pregnancy stress with cortisol levels in pregnancy. Twenty-nine of these included measures of stress in pregnancy only, and 8 included measures of stress that occurred in both the proximal preconception period and pregnancy that were combined to encompass preconception and pregnancy stress. The most common measure of stress was perceived stress. Most studies included measured multiple forms of stress (e.g., chronic stressors, life events, perceived stress) that were either examined separately or as a composite.

#### Diurnal Cortisol.

Levels of stress during pregnancy were associated with differences in the cortisol awakening response in two studies, but the direction of the effect varied. Higher perceived stress in early pregnancy was associated with a greater cortisol awakening response in the third trimester (30–32 weeks) (*n* = 100) (Urizar et al., 2019). Conversely, a smaller study of 30 participants found that higher perceived stress was associated with a more blunted cortisol awakening response in the third trimester (Simon et al., 2016); external stressors (home hardship, stressful life events, and food security) and discrimination proximal to and during pregnancy were not associated with the cortisol awakening response. Two studies reported null associations of stress in pregnancy (perceived stress, chronic stress) with the cortisol awakening response (Bolten et al., 2011; la Marca-Ghaemmaghami et al., 2021).

Three studies tested associations of stress during pregnancy with diurnal cortisol slope. There was a significant cross-sectional association between perceived stress and a flatter diurnal slope in mid pregnancy (26–28 weeks; *n* = 92) (Smew et al., 2018) whereas another study reported null associations of perceived stress with cortisol slope across pregnancy (*n* = 100) (Urizar et al., 2019). Among Black but not Hispanic participants, higher cumulative stress in pregnancy (composite of interpersonal violence, discrimination, negative life events) assessed in mid-pregnancy was associated with a flatter diurnal slope in mid-pregnancy (~25 weeks) (*n* = 200; Suglia et al., 2010).

#### Tonic Cortisol: Salivary.

Five studies reported cross-sectional associations between stress during pregnancy with tonic cortisol levels based on single measures of cortisol. Higher perceived stress was associated with lower levels of morning cortisol in mid pregnancy (26–28 weeks; *n* = 92) (Smew et al., 2018). Higher cumulative stress was also cross-sectionally associated with lower morning cortisol among Black (*n* = 68) but not Hispanic participants (*n* = 132) in mid pregnancy (Suglia et al., 2010). Furthermore, higher perceived stress and exposure to violence in pregnancy were associated with higher afternoon cortisol levels at 18–39 weeks gestation (*n* = 147) (Valladares et al., 2009). Finally, both stressful life events and pregnancy related stress were cross-sectionally, but not prospectively, associated with higher evening cortisol levels in early pregnancy (median=14 weeks) and late pregnancy (median=30 weeks) (*n* = 650) (Obel et al., 2005).

Six studies reported null associations between measures of stress in pregnancy and single measures of cortisol in the morning (Cesta et al., 2018; Harville et al., 2009; Pluess et al., 2012; Shea et al., 2007; Urizar et al., 2004), afternoon (Voegtline et al., 2013), and evening (Cesta et al., 2018; Urizar et al., 2004). These studies variously operationalized stress using measures of perceived stress, life events in the past year, recent stressors and daily stress, stressful life events in the past 6 months, and pregnancy hassles.

One study used an ecological momentary assessment study design (*n* = 152) to examine within- and between-person associations of momentary, daily, and monthly perceived stress with momentary, daily, and monthly salivary cortisol levels during early pregnancy (M=12.3 weeks), mid pregnancy (M= 19.3 weeks), and late pregnancy (M=28.7 weeks) (Lazarides et al., 2020).^3^ At the within-person level, higher levels of momentary and daily perceived stress were associated with higher levels of momentary and daily cortisol output, respectively. That is, in moments and days when individuals reported higher stress than their average levels, their cortisol levels for that moment and day were also higher. Perceived stress was not associated with cortisol at a between-person level. Two studies reported null associations of recent stressors, daily stress, and perceived stress in pregnancy with total cortisol output (Bolten et al., 2011; Harville et al., 2007).

#### Tonic Cortisol: Hair.

Two studies reported significant between-persons associations between stress during pregnancy and hair cortisol (Hoffman et al., 2016; Kalra et al., 2007). Perceived stress in early pregnancy (16–18 weeks) was associated with higher hair cortisol in mid pregnancy (28–30 weeks; *n* = 90) (hair samples collected in early, mid, and late pregnancy and segmented to reflect hair cortisol in the past three months; Hoffman et al., 2016). There was also a significant cross-sectional association between perceived stress measured in the late first or early second trimester and higher hair cortisol (*n* = 25) (hair samples collected at the end of first trimester or beginning of the second trimester and reflecting the previous 1–1.5 months; Kalra et al., 2007). Another study evaluated the within- and between-person associations of stress with hair cortisol from mid to late pregnancy (12–37 weeks) through 5–8 months postpartum (*n* = 90). Within-person fluctuations in stress were coupled with higher hair cortisol such that when individuals reported higher levels of stress in the past 6 months compared to their own average level of stress, hair cortisol were also higher (samples collected at 12–37 weeks gestation, 3–8 weeks postpartum, and 5–8 months postpartum that reflected hair cortisol concentrations in the past 5 months; King et al., 2022). The between-person association between stress and hair cortisol was not statistically significant.

Most studies reported null associations between proximal preconception or pregnancy stress with hair cortisol (*n* = 8) when stress was operationalized as changes in stress from the second to third trimester (Scharlau et al., 2018), stressful life events (Galbally et al., 2019), episodic stress (Conradt, Shakiba et al., 2020), chronic stress (Braig et al., 2016; Conradt, Shakiba et al., 2020), perceived stress (Duffy et al., 2018; Musana et al., 2020), economic stressors and exposure to violence (Orta et al., 2019), or a composite measure of stress (Kramer et al., 2009).

## 5. Lifetime stress

Participants retrospectively reported on the number of stressors that occurred over the course their life (i.e., from early life through timing of stress assessment in pregnancy) without specifying when in life the stressor occurred in four studies (Bosquet Enlow et al., 2017; Campbell et al., 2019; Ghosn et al., 2019; Schreier et al., 2016). Lifetime stress was either dichotomized as having a history of lifetime stress vs. not, or continuously evaluated based on the total number of lifetime stressors.

### Tonic Cortisol: Saliva.

Three studies evaluated associations of lifetime stress with single measures of cortisol. Individuals with a history of lifetime interpersonal trauma (e.g., physical attack, hit with something that hurt them) had higher afternoon cortisol levels in late pregnancy (M=29 weeks) compared to individuals with no trauma history (*n* = 455; Campbell et al., 2019). There were no differences in morning or evening cortisol levels depending on trauma history. In a smaller study of 101 participants, lifetime trauma history (stressors that met DSM-IV Criterion A) was associated with higher morning cortisol levels (10 am-noon) in late pregnancy (38 weeks) compared to participants with no trauma history (Ghosn et al., 2019).

### Tonic Cortisol: Hair.

Two studies included individuals enrolled in a prospective pregnancy cohort tested associations of lifetime stressors and hair cortisol. Lifetime traumatic events (stressors that met DSM-IV Criterion A) assessed in mid pregnancy were associated with higher hair cortisol across pregnancy (*n* = 180; hair samples collected just after delivery; Schreier et al., 2016). In stratified analyses by race/ethnicity, lifetime traumatic events were only associated with higher hair cortisol in Black individuals, but not White or Hispanic individuals. Another study of 195 participants in this cohort reported null associations of lifetime stressors and third trimester hair cortisol (hair samples collected just after delivery; Bosquet Enlow et al., 2017).^4^ Differences in results across reports in this cohort could be due to differences in analytic methods, covariates, or operationalization of lifetime stressors.

### 5.1. Interactive effects

Ten studies included in the present review also evaluated interactive effects. Significant main effects are presented above by developmental period if they were not qualified by a significant interaction. Eight studies reported that there was a significant moderated effect of stress on at least one measure of cortisol in pregnancy as a function of childhood stress, the social environment, and environmental pollution (Bowers et al., 2018; Bublitz et al., 2014, 2016; Bublitz and Stroud, 2013; Epstein et al., 2020; Schreier et al., 2015a; Swales et al., 2018; Thomas et al., 2018a). Two studies reported null interactive effects across all measures of cortisol (Stephens et al., 2021; Thomas et al., 2018b). Further detail on null interactive effects is presented in Table 3.

#### Interactive Effects: Childhood by Prenatal Stress.

The most frequent interactive effect tested was the interaction of childhood stress and pregnancy stress in predicting cortisol levels.

Five studies found that the association between stress during pregnancy and measures of cortisol in pregnancy varied depending on the level of childhood stress (Bowers et al., 2018; Bublitz et al., 2016; Bublitz and Stroud, 2013; Epstein et al., 2020; Swales et al., 2018) and two studies found that there was no significant interactive effect of childhood and pregnancy stress on cortisol (Stephens et al., 2021; Thomas et al., 2018b). Of those reporting a significant interactive effect, most studies found that there was a stronger positive association between stress and cortisol in pregnancy among individuals who experienced childhood stress, with one exception (Bublitz et al., 2016).

#### Diurnal Cortisol.

Childhood stress (< 18 years) modified the association of adulthood stress (> 18 years through pregnancy) with indices of diurnal cortisol in mid-pregnancy (M=26.5 weeks; *n* = 58; Epstein et al., 2020). More stressors in adulthood were associated with a flatter cortisol slope among individuals who experienced fewer stressors in childhood but associated with a steeper cortisol slope among individuals who experienced more stressors in childhood. Moreover, more stressors in adulthood were associated with a blunted cortisol awakening response among individuals who experienced more childhood stressors but not among individuals who experienced fewer childhood stressors.

#### Tonic Cortisol: Saliva.

History of childhood sexual abuse modified associations between prior day pregnancy stress in mid pregnancy and cortisol levels 30 min after waking the following day (*n* = 41) (Bublitz and Stroud, 2013). Prior day stress was associated with higher levels of cortisol 30 min after waking among individuals who had experienced childhood sexual abuse, but not among individuals who had not during mid pregnancy (M=28 weeks). A pilot study (*n* = 17) also found that childhood stress modified the association between momentary stress (assessed by text message 4 times a day) and cortisol levels approximately 30 min later in mid pregnancy (M=27 weeks) and late pregnancy (M=34 weeks) (Bublitz et al., 2016). Higher momentary stress was associated with higher levels of cortisol at the same moment among individuals without a history of childhood maltreatment but was associated with lower levels of cortisol at the same moment among individuals with a history of childhood maltreatment.

#### Tonic Cortisol: Hair.

Two studies found that childhood stress modified associations between adulthood stress and hair cortisol. In a sample of 30 pregnant individuals, adverse childhood experiences moderated the association between perceived stress in pregnancy and hair cortisol (hair samples collected in mid pregnancy reflecting the prior three months; Bowers et al., 2018). Perceived stress in pregnancy was associated with higher hair cortisol in mid pregnancy (weeks gestation not specified) among individuals who experienced two or more adverse childhood events, but not among individuals who experienced less than two adverse childhood events. In another study (*n* = 90), both childhood and adulthood trauma independently predicted higher hair cortisol in mid-pregnancy (M=28.6 weeks; hair samples collected in mid to late pregnancy reflecting the three prior months), but these main effects were qualified by an interaction between adulthood and childhood trauma in predicting hair cortisol. Specifically, the positive association between adulthood trauma and hair cortisol was stronger among individuals who had experienced at least one traumatic event in childhood compared to individuals who had not (Swales et al., 2018).

#### Interactive Effects: Childhood Stress by Prenatal Social Environment.

Two studies found that the association between childhood stress and diurnal cortisol in pregnancy varied as a function of the prenatal social environment. Childhood sexual abuse was associated with increases in the cortisol awakening response from the second to third trimester among individuals with poorer family functioning in pregnancy, but not among individuals with better family functioning in pregnancy (*n* = 185) (Bublitz et al., 2014). More adverse childhood experiences were associated with a steeper cortisol slope in late pregnancy (27–37 weeks) among individuals who reported low levels of social support, but not among those who reported high levels of social support (*n* = 243; Thomas et al., 2018a).

#### Interactive Effects: Prenatal Stress by Environmental Pollution.

One study evaluated the role of environmental pollution, as measured by mercury exposure, in modifying the association between stressful life events in pregnancy and the cortisol awakening response in the second trimester (M=19.5 weeks) (Schreier et al., 2015b). Stressful life events were associated with a blunted cortisol awakening response among individuals exposed to high levels of pollution, but not among individuals exposed to low levels of pollution (*n* = 732).

## 6. Discussion

Many changes in the HPA axis during pregnancy such as increases in CRH, ACTH, and cortisol from early to late pregnancy are essential to facilitating fetal development, alterations in the maternal brain, and parturition. However, HPA axis dysregulation and elevated levels of cortisol are hypothesized to be a central biological pathway linking maternal stress before and during pregnancy to adverse maternal and child health. The current scoping review is the first of its kind to identify, characterize, and summarize literature on the associations of maternal stress before and during pregnancy with cortisol in pregnancy as measured by hair and saliva. Consistent with established theory on stress, we conceptualized stress as a process involving stressors and appraisals (Lazarus and Folkman, 1984).

We identified 48 studies that were eligible for inclusion in the review. Salivary cortisol was the most common measure of cortisol, but an increasing number of studies in recent years include measures of hair cortisol concentrations. Most studies measured stress in the proximal preconception period (within one year of pregnancy) or during pregnancy. Consistent with life course approaches, results of the current review indicate that the associations between stress and cortisol in pregnancy vary as a function of the developmental timing of stress exposure as well as interactions of risk and protective factors across the lifespan. Specifically, there were more consistent associations between childhood stress and cortisol in pregnancy compared to proximal preconception or pregnancy stress. The studies we reviewed involved mainly healthy, low-risk samples. In addition, we did not evaluate associations of cortisol with fetal or child outcomes (reviewed elsewhere in Zijlmans et al., 2015). Thus, these results do not suggest any effects of fetal or child development, nor clinically significant changes in maternal prenatal cortisol. Below, we summarize findings, identify research gaps, and offer recommendations for future research.

## 7. Overview of primary findings

### 7.1. Childhood stress

Childhood stress predicted several cortisol indices in pregnancy, broadly consistent with findings in pregnant (Epstein et al., 2021) and non-pregnant adults (Bunea et al., 2017; Fogelman and Canli, 2018). Consistent with the prior review in pregnant adults (Epstein et al., 2021), higher levels of childhood stress were associated with a greater cortisol awakening response in pregnancy. The current review provides additional evidence that childhood stress is associated with changes in diurnal patterns over the course of pregnancy, namely less flattening of the diurnal slope and less attenuation of the cortisol awakening response across pregnancy. The associations between childhood stress and diurnal cortisol did not appear to differ as a function of gestational age at time of cortisol sample collection; that is, childhood stress showed consistent associations with the cortisol awakening response in early, mid, and late pregnancy and with changes in diurnal indices from early to late pregnancy. These results are also consistent with findings in non-pregnant samples indicating that both the strength and the direction of the association of childhood stress with cortisol levels vary depending on several factors, including the measure of cortisol (Bunea et al., 2017; Fogelman and Canli, 2018). For example, results of tests of associations between childhood stress and tonic levels of cortisol were less consistent. For example, only 2 of 8 studies found that childhood stress was significantly associated with greater total cortisol output or hair cortisol and the direction of effects varied across studies. The inconsistency in associations between childhood stress and tonic cortisol could also be due in part to confounding factors known to influence single measurements of salivary cortisol such as recent physical activity and food or caffeine intake (e.g., Dahlgren et al., 2009; Hruschka et al., 2005; Segerstrom et al., 2014). Taken together, these findings suggest that childhood stress may shape diurnal cortisol patterns during pregnancy, but not overall cortisol output or tonic levels.

Associations of childhood stress with maternal prenatal diurnal cortisol patterns may indicate developmental programming of the HPA axis (Danese and McEwen, 2012). Developmental programming refers to the process whereby stress exposure during developmental periods characterized by rapid neuroendocrine development and heightened sensitivity to the social environment, such as childhood, show particularly robust associations with cortisol regulation (Gunnar and Howland, 2022). During these developmental periods, the HPA axis is particularly sensitive to stress exposure, including repeated, intense, or prolonged stress, such that childhood experiences of stress can influence HPA axis feedback mechanisms across the lifespan and during pregnancy (Danese and McEwen, 2012; Steine et al., 2020). Notably, the results of the present review indicate that the effects of stress during childhood on HPA axis regulation in pregnancy may be stronger than experiences of stress proximal to or during pregnancy.

There are several pathways through which childhood stress may influence diurnal cortisol regulation during pregnancy. Long-term sleep disturbances may be a critical factor contributing to variation in the cortisol awakening response and diurnal slope. Sleep disturbances are well-documented among non-pregnant adolescents and adults who experienced high levels of stress during childhood (see Fuligni et al., 2021 for a review). Emerging evidence also suggests that childhood stress predicts sleep disturbances during pregnancy (Foss et al., 2022; Nevarez-Brewster et al., 2022). The diurnal rhythm, particularly the cortisol awakening response, is superimposed upon the circadian cycle and, therefore, intimately linked with sleep (Wilhelm et al., 2007). For example, more sleep problems and shorter sleep duration have been associated with a greater cortisol awakening response (Kuhlman et al., 2019; Vargas and Lopez-Duran, 2014). Dysregulation of diurnal cortisol patterns in pregnancy could also occur through epigenetic pathways. Childhood stress has been associated with differential methylation of glucocorticoid receptor genes and gene expression that may serve a key role in regulation of the HPA axis and diurnal rhythm (Koss and Gunnar, 2018; Szyf and Bick, 2013; Tyrka et al., 2016). Further investigation on the biobehavioral pathways linking childhood stress to diurnal cortisol rhythms in pregnancy could help to inform intervention targets, particularly with a focus on how different types of childhood stress could operate through distinct biobehavioral pathways (Bublitz and Stroud, 2012, 2013; Thomas et al., 2018b).

The extent to which childhood stress predicted diurnal cortisol varied as a function of the prenatal social environment. High childhood stress was associated with a lack of the flattening in diurnal slope and attenuation of cortisol awakening response that are expected during pregnancy, but only among individuals who had low social support or poor family functioning in pregnancy, respectively (Bublitz et al., 2014; Thomas et al., 2018a). Notably, however, individuals who experienced high levels of childhood stress showed the expected changes in diurnal patterns over pregnancy if they reported high levels of social support or better family functioning during pregnancy. Collectively, these results suggest that a supportive social environment during pregnancy may ameliorate and/or recalibrate the effects of childhood adversity on HPA axis regulation, offering a potential area of intervention (Davis and Narayan, 2020; Gunnar and Howland, 2022; Thomas et al., 2018a).

### 7.2. Proximal preconception and pregnancy stress

Associations of proximal preconception and pregnancy stress with cortisol in pregnancy were less consistent than associations between childhood stress and cortisol in pregnancy. Most of these studies reported null associations and the studies that did report significant associations of proximal preconception and pregnancy stress with cortisol levels were inconsistent in the patterns of findings across studies. For example, one study reported that greater stress during pregnancy predicted a smaller cortisol awakening response while another reported that prenatal stress predicted a larger response. Two additional studies reported null associations. In a few studies, there was some evidence of an association between higher levels of stress in the proximal preconception period and in pregnancy with a flattened diurnal slope (*n* = 2), lower morning levels of cortisol (*n* = 2), and higher afternoon or evening levels of cortisol (*n* = 3) in pregnancy. However, associations between proximal preconception and pregnancy stress with hair cortisol were mostly null (*n* = 8) with 2 exceptions. Importantly, two of the studies reported significant within-person but not between-person associations (King et al., 2022; Lazarides et al., 2020). Specifically, on occasions when individuals reported higher levels of stress than their average levels of stress, their cortisol levels were also higher than their average levels; however, higher levels of stress were not associated with higher cortisol across individuals. Therefore, the extent to which stress is associated with cortisol may depend on what level of stress is “typical” for an individual and an individual’s experiences of stress.

The current review included studies that measured proximal preconception and pregnancy stress based on stressors and stress appraisals. Prior evidence in non-pregnant samples demonstrates that different forms of stress may show stronger associations with cortisol levels (e.g., Dickerson and Kemeny, 2004). Here, our ability to compare whether different forms of stress (i.e., stressors vs. stress appraisals) were more consistently associated with cortisol indices was somewhat limited by the use of different stress measures across studies and use of composite variables that included stressors and perceptions (see Table 3 for detail on operationalizations). Overall, the present findings did not suggest greater consistency in associations between stressors and prenatal cortisol as compared to stress appraisals and prenatal cortisol; that is, stressors were not more consistently or more strongly associated with prenatal cortisol than stress appraisals (e.g., perceived stress). There was also some inconsistency across studies measuring one type of stress. For example, one study found that perceived stress was associated with greater third trimester cortisol awakening responses (Urizar et al., 2019), whereas another found that perceived stress was associated with a blunted cortisol awakening response in the third trimester (Simon et al., 2016). Notably, in the two studies reporting significant within-person associations between stress and cortisol, one study measured perceived stress (Lazarides et al., 2020) and the other measured stressful life events (King et al., 2022), suggesting that deviations from what is “typical” for that individual in regard to both stressors and perceptions may predict prenatal cortisol.

Several investigations reported that the extent to which stress proximal to or during pregnancy predicted cortisol levels depended on the level of childhood stress, indicating the utility of a life course perspective for understanding HPA axis activity during pregnancy. For example, there was stronger positive association of stress in pregnancy with hair and morning salivary cortisol among individuals who experienced higher levels of childhood stress (Bowers et al., 2018; Bublitz and Stroud, 2013; Swales et al., 2018). Furthermore, adulthood stress was associated with dysregulation of diurnal patterns across pregnancy among those with a history of more childhood stressors, but not among those with a history of fewer childhood stressors (Epstein et al., 2020). Thus, early life adversity may sensitize the HPA axis to dysregulation after later stress. Overall, these findings suggest cumulative effects of childhood stress and prenatal stress on HPA axis regulation in pregnancy and indicate that childhood stress calibrates stress responses across the lifespan, consistent with the Adaptive Calibration Model. Finally, a few studies found that the association of proximal preconception and pregnancy stress and prenatal cortisol varied by race/ethnicity and were stronger among Black/African American participants than Hispanic/Latina or White participants (e.g., Schreier et al., 2015a; Suglia et al., 2010). Future investigation of how understudied forms of stress that pregnant people of color are disproportionately exposed to, including systemic racism, discrimination, historical trauma, and acculturation, influence prenatal HPA axis regulation is necessary (Conradt, Carter et al., 2020).

## 8. Research gaps and directions for future research

The results of this scoping review suggest several limitations in the current literature that may contribute to mixed results. Based on these limitations, we provide recommendations for future research, summarized in Table 4.

First, the operationalization of stress during the proximal preconception period and pregnancy varied in nature, timing, duration, and whether measures of stress were examined separately or in composite scores. Although these operationalizations reflect that the stress process involves both exposures that vary across timescales and perceptions of exposures, the extent to which stress predicts cortisol levels and stress physiology more generally may depend on how stress is operationalized (e.g., exposure vs. appraisals, timing, type) (Dickerson and Kemeny, 2004; Epel et al., 2018; O’Donnell and Meaney, 2017). For example, in non-pregnant samples, the associations between stress and both cortisol reactivity and hair cortisol vary depending on the type and timing of the stress and adversity (Dickerson and Kemeny, 2004; Khoury et al., 2019). As compared to assessments of stress during the proximal preconception period or in pregnancy that used many different stress measures, retrospective reports of childhood stress often used the same measures across studies, namely the Adverse Childhood Experiences Questionnaire and the Childhood Trauma Questionnaire. Thus, it is plausible that the childhood stress findings in the current review are more consistent in part because the same measures were used across studies. Of note, however, several studies in the current review also reported the association between childhood stress and cortisol in pregnancy varied as a function of the type (e.g., sexual abuse vs. emotional abuse; Bublitz et al., 2014; Schreier et al., 2015a). Specifically, several studies reported stronger associations between childhood sexual abuse and HPA axis regulation in pregnancy compared to other types of childhood stress (e.g., Bublitz et al., 2016; Bublitz and Stroud, 2013; Stephens et al., 2020).

Recent work in perinatal samples has found that the association between maternal stress and length of gestation varies as a function of the type of stress. Specifically, stress appraisals were inversely associated with length of gestation, whereas there was a curvilinear association between stress exposures and length of gestation, suggesting that different forms of stress show distinct patterns of associations with birth outcomes (Mahrer et al., 2021). Taken together, these findings underscore a need for more precision in conceptualization and measurement of stress prior to and during pregnancy to better understand associations with maternal-fetal health. Further investigation of whether the association of stress with cortisol levels vary as a function of the type of stress may help to elucidate links with maternal cortisol levels in pregnancy and maternal-fetal health.

Different types of stress (chronic stressors, acute stressors, perceived stress) and stress across developmental periods (childhood, adulthood, pregnancy) may also act synergistically to influence cortisol levels in pregnancy. Results of the current review provide support for recent conceptual models positing that the effects of stress processes on health depend on interactions between individual and environmental factors that unfold across development and are influenced by both history of and current stress (Epel et al., 2018). Across several studies, the associations between stress and cortisol levels varied depending on the timing of exposure and factors such as social support and environmental pollution (Bublitz and Stroud, 2013; Schreier et al., 2016; Thomas et al., 2018a). Future investigations should continue to consider multi-level, interactive processes through which stress influences prenatal stress physiology.

The measurement of cortisol also varied over studies. Although most studies on salivary cortisol had measurements over several consecutive days and at multiple timepoints during pregnancies, others included measurements on only one day or only one sample per day. HPA axis activity changes substantially from early to late pregnancy and therefore careful consideration of timing of sample collection and multiple measurements of cortisol across gestation is necessary (Entringer et al., 2010; Howland et al., 2017). Single measures can be problematic because tonic salivary cortisol is influenced by a range of factors, including food/caffeine intake and time of day, and consequently can vary up to 70% within individuals day-to-day (Dahlgren et al., 2009; Hruschka et al., 2005). Prior research shows that three days of samples are needed to generate reliable estimates of cortisol levels, with more sampling days necessary for diurnal measures like cortisol slope (Segerstrom et al., 2014). Thus, assessment of cortisol across multiple days in pregnancy with careful consideration to provide clear instructions regarding food intake and sample timing is critical in future research.

Careful consideration of the timing of both cortisol and stress assessments are especially important study design components in pregnancy given that maternal perceptions of and responses to stress change across pregnancy along with indices of cortisol (de Weerth et al., 2005; Entringer et al., 2010; Howland et al., 2017). For example, a few studies found that there were significant associations between stress and cortisol at certain gestational timepoints (e.g., early pregnancy), but not others (e.g., late pregnancy; Thomas et al., 2018a). Most studies in the current review use measures of stress or cortisol taken on a single occasion, most often in mid pregnancy, precluding examination of whether the association between stress and cortisol differed across gestational timepoints. This may be a possible avenue for future research. Repeated assessments of both stress and cortisol across pregnancy may be necessary to better understand the links between maternal stress and cortisol over the course of pregnancy.

The results of the current review also suggest there may be analytic reasons for mixed results. First, there was substantial variation in whether covariates were included, and if so, which ones were included. Statistically controlling for factors with demonstrated links to cortisol in pregnancy (e.g., gestational age, corticosteroid use, time of cortisol collection) in future research could help to reduce confounding and promote comparison across studies (Dahlgren et al., 2009; Hruschka et al., 2005; Segerstrom et al., 2014). Controlling for gestational age at the time of cortisol assessment is particularly important given the systematic increases in tonic cortisol levels and diurnal cortisol indices over the course of pregnancy (Howland et al., 2017). Moreover, most studies did not control for psychological distress in pregnancy whereas others did and reported effects of stress on cortisol independently of psychological distress (Thomas et al., 2018b). It was beyond the scope of the current review to evaluate studies on psychological distress, or those that included psychological distress in composite measures of stress (see Khoury et al., 2022; Mustonen et al., 2018; Seth et al., 2016 for recent reviews on this topic). Future reviews that include studies examining effects of both stress and psychological distress, including independent and interactive effects, may help elucidate factors that shape prenatal stress physiology.

Most studies included in the current review examined between-persons associations of stress with cortisol levels (i.e., across individuals); however, analytic methods that adopt person-centered approaches or parse variability at an individual-level and across individuals may help to clarify links between stress and cortisol. For example, person-centered approaches can identify subpopulations that show distinct patterns of change using longitudinal data. One recent study that used general growth mixture modeling found that maternal psychological distress in pregnancy predicted 3 different plasma cortisol trajectories over pregnancy (Peterson et al., 2020), highlighting the utility of person-centered approaches in elucidating links between maternal distress and cortisol in pregnancy. Multilevel models may also be employed in future studies to explain variation both at an individual-level and across individuals. In general, multilevel modeling has been recommended to evaluate associations between cortisol and factors such as stress, as it can lead to a significant improvement in the precision of person-level estimates and help account for individual variability in cortisol levels across days (Hruschka et al., 2005). Consistent with these recommendations, two key studies in this review found that there were within-persons but not between-persons associations of stress with cortisol using multilevel modeling (King et al., 2022; Lazarides et al., 2020). Longitudinal assessments of stress and cortisol over pregnancy paired with multilevel modeling or person-centered analytic approaches may help to clarify the associations between stress and cortisol in pregnancy by disaggregating within and between person effects that would otherwise be confounding.

The stress-sensitive nature of the HPA axis and cortisol levels has led many to hypothesize that cortisol levels in pregnancy serve as a primary pathway linking maternal stress to maternal-fetal health (Cottrell and Seckl, 2009). Based on the results of this review, however, associations between stress and cortisol levels in pregnancy are inconsistent and depend on the developmental timing as well as interactions of risk and resilience factors across development. Other indicators of HPA axis functioning like cortisone, pCRH, and dehydroepiandrosterone (DHEA) could be examined in future work. For example, one study included in the current review reported null associations between stress and hair cortisol but found that stress was associated with changes in hair cortisone (inactive metabolite of cortisol) and the ratio of hair cortisone to hair cortisol (Scharlau et al., 2018). pCRH is also sensitive to maternal stress signals in pregnancy and may have a more direct effect on fetal development and birth outcomes than cortisol given its placental-fetal origin (Rinne et al., 2022; Sandman, 2018). Further assessment of the coordination of the HPA axis with other physiological systems that support maternal-fetal health is also warranted. In particular, indicators of immune system function may be important to examine independently or in coordination with the HPA axis (neuroendocrine-immune coupling; Hantsoo et al., 2019; Riis et al., 2020).

## 9. Conclusions

Altered HPA axis functioning during pregnancy is a potential mechanism through which maternal stress before concpetion and during pregnancy influences maternal-fetal health. The present scoping review identified and summarized literature on the associations between stress in childhood, the proximal preconception period, and pregnancy with cortisol levels in pregnancy. Overall, the associations between stress and cortisol levels varied as a function of the developmental timing of stress, with more consistent associations with stress during childhood than stress during the proximal preconception period or pregnancy, and several moderators. Future studies that consider stress across the lifespan as emergent and interactive, include multiple measurements of cortisol over pregnancy and statistically control for confounders, and employ person-centered analytic approaches may further elucidate the links between stress and cortisol levels in pregnancy.

## Figures and Tables

**Table 1:** Search Terms.

Concept	Search Terms

Stress	(“life events” OR “violence” OR “discrimination” OR “abuse” OR “trauma” OR “adversity” OR “stress”)
Pregnancy	AND (“pregnancy” OR “pregnant woman” OR “pregnant” OR “prenatal” OR “antenatal” OR “perinatal” OR “pregnancies”)
Cortisol	AND (“cortisol” OR “hypothalamic pituitary adrenal” OR “hypothalamic-pituitary-adrenal” OR “HPA” OR “glucocorticoid” OR “neuroendocrine” OR “stress response”)
Databases	PubMed, PsycINFO, CINAHL
Filters	Humans, Adults
Years Included	Unrestricted through January 2022

*Note.* Title and abstract search.

**Table 2:** Synthesis of primary findings reporting an association between stress and cortisol levels.

Developmental period	Measure of cortisol	Findings	Evidence

**Childhood stress**	*Diurnal*	(a) Greater cortisol awakening response (*n* = 5) (b) Increasing cortisol awakening response across pregnancy (*n* = 1) (c) Less flattening of the diurnal cortisol slope across pregnancy (*n* = 2)	(a) Epstein et al. (2020);Stephens et al. (2021);Thomas et al. (2018a);Thomas et al. (2018b); Thomas-Argyriou et al. (2021) (b) Bublitz and Stroud (2012) (c) Thomas et al. (2018b);Stephens et al. (2021)
	*Tonic: Saliva*	(a) Lower waking cortisol levels (*n* = 1) (b) Higher wake + 30 cortisol levels (*n* = 1) (c) Higher evening cortisol levels (*n* = 1)	(a) Shea et al. (2007) (b) Thomas et al. (2018b) (c) Jairaj et al. (2020)
	*Tonic: Hair*	(a) Higher hair cortisol concentrations (*n* = 1)^a^ (b) Lower hair cortisol concentrations (*n* = 2)	(a) Schreier et al. (2015a) (b) Bowers et al. (2018);Nyström-Hansen et al. (2019)
**Proximal preconception and/or pregnancy stress**	*Diurnal*	(a) Greater cortisol awakening response (*n* = 1) (b) Lower cortisol awakening response (*n* = 1) (c) Flatter slope (*n* = 2)^a^	(a) Urizar et al. (2019) (b) Simon et al. (2016) (c) Suglia et al. (2010);Smew et al. (2018)
	*Tonic: Saliva*	(a) Lower morning cortisol levels (*n* = 2) (b) Higher afternoon cortisol levels (*n* = 1) (c) Higher evening cortisol levels (*n* = 1) (d) Higher momentary cortisol levels and daily cortisol output (*n* = 1)^b^	(a) Suglia et al. (2010);Smew et al. (2018) (b) Valladares et al. (2009) (c) Obel et al. (2005) (d) Lazarides et al. (2020)
	*Tonic: Hair*	(a) Higher hair cortisol concentrations (*n* = 3)^b^	(a) Kalra et al. (2007);King et al. (2022);Hoffman et al. (2016)
**Lifetime stress**	*Diurnal*	—	—
	*Tonic: Saliva*	(a) Higher morning cortisol (*n* = 1) (b) Higher afternoon cortisol (*n* = 1) s	(a) Ghosn et al. (2019) (b) Campbell et al. (2019)
	*Tonic: Hair*	(a) Higher hair cortisol (*n* = 1)^a^	(a) Schreier et al. (2016)
**Moderated effects**	*Diurnal*	(a) Adulthood stress associated with a flatter cortisol sleep among individuals who experienced low childhood stress but associated with a steeper cortisol slope among individuals who experienced higher stress in childhood (*n* = 1) (b) A more severe history of childhood sexual abuse and poorer family functioning in pregnancy predicted increases cortisol awakening response from the second to the third trimester compared to individuals with a less severe childhood sexual abuse history and better family functioning (*n* = 1) (c) More adverse childhood experiences were associated with a steeper cortisol slope among individuals who reported low levels of social support but not among those who reported high levels of social support (*n* = 1)	(a) Epstein et al. (2020) (b) Bublitz et al. (2014) (c) Thomas et al. (2018a)
	*Tonic: Saliva*	(a) Stress in pregnancy associated with higher levels of wake + 30 cortisol levels among individuals who had experienced childhood sexual abuse, but not those who had not (*n* = 1) (b) Momentary stress associated with higher levels of cortisol among individuals without a history of childhood maltreatment but associated with lower levels of cortisol among individuals with a history of childhood maltreatment (*n* = 1) (c) Stressful life events associated with a blunted cortisol awakening response among individuals who with high mercury levels but not low mercury levels (*n* = 1)	(a) Bublitz and Stroud (2013) (b) Bublitz et al. (2016) (c) Schreier et al. (2015b)
	*Tonic: Hair*	(a) Adulthood and pregnancy stress was more strongly, positively associated with hair cortisol among individuals who had experienced higher levels of childhood stress (*n* = 2)	(a) Bowers et al. (2018);Swales et al. (2018)

*Note.* Table summarizes studies reporting associations between stress and measures of cortisol; null results are presented in Table 3 in further detail. No studies evaluated associations of lifetime stress with diurnal cortisol indices.

a In stratified analyses, the association between stress and cortisol was only significant among Black participants.

b Stress was associated with cortisol at the within-persons, but not between-persons level.

**Table 3:** Synthesis of studies included in the scoping review (n = 48), organized by developmental period and alphabetically within each developmental period.

Reference	Study setting	Sample characteristics	Stress measure and timing of assessment	Cortisol measure and timing of assessment	Type of statistical analysis testing associations of stress and cortisol; covariates included in models (if applicable)	Summary of primary results

**Studies including measures of childhood stress (*n* = 7)**						
Bublitz and Stroud (2012)	135 pregnant individuals enrolled in a longitudinal study of the effects of maternal mood on fetal and infant development (Behavior and Mood in Mothers, Behavior in Infants [BAMBI])	M age = 26 years (SD = 6 years); 42% Non Hispanic White, 23% Hispanic, 14% Non Hispanic Black, 12% multiracial, 5% Asian, 4% other; M_income_ = $30–39k; 36%– 42% married (by child abuse status)	**Childhood stress** Adverse Childhood Experiences Questionnaire 15-item version Retrospective report in mid-pregnancy (M=24 weeks, range = 20–26 weeks)	**Salivary cortisol** Waking, 30 min after waking, and at bedtime over 3 consecutive days at 1–3 timepoints in the second and third trimester (M_1_ = 24 weeks, range = 20–26 weeks; M_2_ = 30 weeks, range = 27–32 weeks; M_3_ = 35 weeks, range = 33–38 weeks) Calculated cortisol awakening response and slope	Hierarchical linear models adjusting for age, race, marital status, parity, gravida, smoking, yearly income, pre-pregnancy BMI, lifetime PTSD, physical illness, gestational diabetes, hypertension, and pre-eclampsia	(+) Individuals with child sexual abuse histories had significantly higher cortisol awakening response at 35 weeks compared to individuals with non-sexual child abuse and no abuse histories. (Ø) No group differences for cortisol slope.
Bublitz et al. (2014)	185 pregnant individuals enrolled in a longitudinal study of the effects of maternal mood on fetal and infant development (Behavior and Mood in Mothers, Behavior in Infants [BAMBI])	M age = 26.5 years (SD = 5.5 years); 43% Non-Hispanic White, 29% Hispanic, 16% Black, 4% Asian, 4% multiracial, 4% other; 47% reported yearly income less than $30,000; 36% married	**Childhood stress** Adverse Childhood Experiences Questionnaire 15-item version Retrospective report at study enrollment **Family functioning in pregnancy** Family Assessment Device (evaluated as moderator) Assessed in the second trimester (~25 weeks)	**Salivary cortisol** Waking, 30 min after waking, and bedtime over three days at 1–3 study visits in the second and third trimesters of pregnancy (M_1_ = 25 weeks, SD_1_ = 4 weeks; M_2_ = 29 weeks, SD_2_ = 1 week; M_3_ = 35 weeks, SD_3_ = 1 week) Calculated cortisol awakening response	Hierarchical linear models adjusting for gestational age at each study visit and timing of waking that included interactive term between childhood stress and family functioning in pregnancy	(⊗) A more severe childhood sexual abuse history was associated with an increasing cortisol awakening response among individuals with poor family functioning but not among individuals with better family functioning (Ø) Other forms of childhood stress (physical abuse, neglect, domestic violence, total scores) were not associated with the cortisol awakening response; social support did not modify associations
Nyström-Hansen et al. (2019)	44 pregnant individuals enrolled in a longitudinal cohort study of mothers with schizophrenia, bipolar disorder, depression, and controls in Denmark (WARM Study)	M age = 30.0 years (SD = 4.47); 97.7% White, 2.3% Asian; Modal educational attainment ‘upper secondary education or more’ (81.9%); 68.2% reported lifetime diagnosis of severe mental illness. 88.6% married or living with their partner	**Childhood stress** Adverse Childhood Experiences Questionnaire (version not specified) Retrospective report in the second or third trimester	**Hair cortisol concentrations** Hair samples collected in the second or third trimester (range = 18.7–38.9 weeks) and segmented to reflect prior four months of pregnancy	Linear regression analyses adjusting for BMI	(−) Adverse childhood experiences were marginally inversely associated with hair cortisol concentrations
Schreier et al. (2015a)	180 pregnant individuals enrolled in a prospective pregnancy cohort designed to examine the role of prenatal stress on stress responses and respiratory health in children (Programming of Intergenerational Stress Mechanisms [PRISM])	M age = 31.01 years (SD = 5.41 years); 45.6% Hispanic, 35.5% White, 18.9% Black; Modal educational attainment ‘less than high school’ (25%)	**Childhood stress** Childhood Trauma Questionnaire. Childhood abuse categorized as present if participants reported any item on emotional abuse, physical abuse, and sexual abuse subscale as occurring between sometimes and very often Retrospective report within two weeks of study enrollment (M=26.9 weeks, SD = 8.1 weeks)	**Hair cortisol concentrations** Hair samples collected near the time of delivery and segmented to reflect all three trimesters (55.6%), the second and third trimesters (36.1%), or third trimester (8.3%) depending on length of hair sample	Mixed-effects analyses of covariance models adjusting for maternal age, education, inhaled corticosteroid use, BMI, and PTSD symptoms Performed in the entire sample and then stratified by race/ethnicity	(+) Individuals who experienced physical/sexual abuse during childhood had higher hair cortisol than women who did not experience childhood abuse. In analyses stratified by race/ethnicity, this association was only significant among Black women, not White or Hispanic women
Schury et al. (2017)	94 pregnant individuals recruited in the maternity ward of the University Hospital Ulm, Germany	M age = 32.5 years (SD = 5.4 years); 88.3% of German origin; 58.1% completed higher school education; 76.6% married	**Childhood stress** Childhood Trauma Questionnaire. Retrospective report within 6 days of giving birth	**Hair cortisol concentrations** Hair samples collected within 6 days of giving birth and segmented to reflect the third trimester of pregnancy	Ordinary least squares models. No covariates in model examining association of childhood trauma with hair cortisol	(Ø) Childhood maltreatment load was not significantly associated with third trimester hair cortisol
Thomas-Argyriou et al. (2021)	248 pregnant individuals enrolled in a prospective pregnancy cohort (Alberta Pregnancy Outcomes and Nutrition [APrON])	M age = 32.3 years (range = 22–43); 86.7% White, 4.4% Latin American, 3.6% Asian, 3.2% Chinese, 2.1% Other; modal household income ‘more than $100,000/year’ (58.9%); 99.2% married or in common law relationships	**Childhood stress** Adverse Childhood Experiences Questionnaire 10-item version Retrospective report of during pregnancy	**Salivary cortisol** Waking, 30 min after waking, 11 am, and 9 pm over two consecutive days in early pregnancy (M=15.1 weeks, SD = 3.5 weeks, range = 6–22 weeks) and late pregnancy (M=32.5 weeks, SD = 1.0 weeks, range = 27–37 weeks) Calculated cortisol awakening response, daytime slope, and total daytime cortisol output	Multiple linear regression adjusting for gestational age at each prenatal assessment, socioeconomic status (composite of income, education, and ethnicity), child sex, and gestational age at birth	(+) Adverse childhood experiences were significantly associated with a greater cortisol awakening response in early pregnancy and in late pregnancy (Ø) Adverse childhood experiences were not associated with cortisol slope or total cortisol output at either timepoint
Thomas et al. (2018a)	243 pregnant individuals enrolled in a prospective pregnancy cohort (Alberta Pregnancy Outcomes and Nutrition [APrON])	M age = 31.3 years (range = 20–42); 82.8% White Caucasian, 4.2% Latin American, 3.8% Chinese, 3.8% Asian, 5.4% Other; modal household income ‘more than $100,000/year’ (57.8%); 98.7% married or in common law relationships	**Childhood stress** Adverse Childhood Experiences Questionnaire 10-item version Retrospective report during pregnancy **Social support in pregnancy** Perception of partner support measured with Social Support Effectiveness Questionnaire (evaluated as moderator) Assessed at each pregnancy visit	**Salivary cortisol** Waking, 30 min after waking, 11 am, and 9 pm over two consecutive days in early pregnancy (M=15.1 weeks, SD = 3.5 weeks, range = 6–22 weeks) and late pregnancy (M=32.5 weeks, SD = 1.0 weeks, range = 27–37 weeks) Calculated cortisol awakening response, daytime slope, and total daytime cortisol output	Regression models adjusting for parity, gestational age, socioeconomic status (composite of income, education, and ethnicity), gestational age at birth, and child sex with an interactive term between childhood stress and social support in pregnancy	(+) Adverse childhood experiences associated with a greater cortisol awakening response in early but not late pregnancy (⊗) Adverse childhood experiences were associated with a steeper slope at low levels of social support (Ø) Adverse childhood experiences were not associated with total cortisol output; social support did not modify this association
**Studies including measures of proximal preconception and pregnancy stress (*n* = 26)**						
Bolten et al. (2011)	81 pregnant individuals recruited to a prospective longitudinal study on child development in Trier, Germany	M age = 31.4 years (SD = 5.3 years); 94.3% White; medium (43.4%) to high (14.5% income); 5.7% less than 8 years of schooling, 57.1% 10–12 years of schooling, 37.2% university degree	**Pregnancy stress** Perceived Stress Scale 14-item version Assessed in mid and late pregnancy	**Salivary cortisol** Waking, 30 min after waking, 45 min after waking, and 60 min after waking over two days in early to mid pregnancy (13–18 weeks) and in late pregnancy (35–37 weeks); Calculated cortisol awakening response	Linear regression models adjusting for age, parity, pre-pregnancy BMI, gestational age at saliva sampling, number of cigarettes smoked, and infant sex	(Ø) Perceived stress was not significantly associated with the cortisol awakening response
Braig et al. (2016)	932 pregnant individuals recruited to a birth cohort study (Ulm SPATZ Health Study) in Ulm, Germany during a hospital stay for delivery	41.5% 30.1–35.0 years, 24.4% 35.1–40.0 years, 22.1% 25.1–30.0 years, 6.5% < 25 years, 5.5% > 40 years; 89.2% German nationality, 1.6% Turkish nationality, 9.2% Other; 58.6% *>* 11 years of education, 31.5% 10–11 years of education, 10.0% less than or equal to 9 years of education	**Pregnancy stress** Trier Inventory of Chronic Stress to assess chronic stress in the prior 3 months Assessed after delivery (median=1.5 days, range = 0–3 days)	**Hair cortisol concentrations** Hair samples collected after delivery (median=1.5 days, range = 0–3 days) that reflected hair cortisol concentrations the third trimester of pregnancy	General linear regression models adjusting for number of people in the household, mode of delivery, season of delivery, smoking status, and BMI	(Ø) Levels of hair cortisol not associated with pregnancy stress
Cesta et al. (2018)	485 individuals undergoing fertility treatment enrolled in a prospective longitudinal study in central Sweden (Uppsala-Stockholm Assisted Reproductive Techniques [UppStART])	M age = 33.8 years. Most educated at the university level	**Pregnancy stress** Perceived Stress Scale 10-item version; Fertility Problem Stress Scales Gestational timing of measurement not specified	**Salivary cortisol** Waking and bedtime. Gestational age at time of collection not specified	Pearson’s correlations	(Ø) Stress was not associated with morning or bedtime cortisol levels
Conradt, Shakiba et al. (2020)	137 pregnant individuals recruited as a part of a longitudinal study on the intergenerational transmission of emotion dysregulation	M age = 29.0 years (SD = 5.2 years); 79.0% Caucasian, 27.2% Hispanic, 9.3% Asian, 6.2% more than one race, 3.1% Native American or Alaskan Native; 1.2% Hawaiian or Pacific Islander; 1.5% African American; 32.1% college graduates, 32.1% completed junior college or technical school; 76.8% married	**Pregnancy stress** UCLA Life Stress Interview assessed episodic and chronic stress in the previous six months Gestational timing of assessment not specified	**Hair cortisol concentrations** Hair samples collected in late pregnancy (M=33 weeks, range = 26–40 weeks) and segmented to reflect the previous six months of pregnancy	Hierarchical linear regression models adjusting for BMI and education	(Ø) Episodic and chronic stress were not associated with hair cortisol concentrations
Duffy et al. (2018)	52 pregnant individuals recruited to a cross-sectional, pilot study	22 participants delivered preterm (M age = 27.7 years, SD = 7.1 years; 54.5% White, 27.3% Black or African American, 18.2% Other; 72.7% Not Hispanic or Latino; 63.6% completed high school or less; 45.5% married) 30 participants delivered at term (M age = 27.4 years, SD = 5.2 years; 66.7% White, 13.3% Black or African American, 16.7% Other, 3.3% Asian; 63.3% Not Hispanic or Latino; 36.7% completed high school or less; 60.0% married)	**Pregnancy stress** Perceived Stress Scale 14-item version to assess perceived stress in the previous month Assessed at delivery	**Hair cortisol concentrations** Hair samples collected at delivery that reflected hair cortisol concentrations in the first trimester, second trimester, and third trimester	Pearson’s correlations and independent samples t-tests	(Ø) No association of perceived stress with hair cortisol
Galbally et al. (2019)	241 pregnant individuals recruited to a selected pregnancy cohort study (Mercy Pregnancy and Emotional Wellbeing Study [MPEWS]) in Australia	M age = 31.4 (SD = 4.7); 88.3% Oceania/European; 8.8% Asian, 2.1% Middle Eastern, 0.8% Aboriginal and Torres Strait Islander; 67.7% completed tertiary university degree; 66.2% married	**Pregnancy stress** Pregnancy Risk Assessment Monitoring System to assess stressful life events. Assessed before 20 weeks of pregnancy and during the third trimester	**Hair cortisol concentrations** Hair samples collected at delivery that reflected hair cortisol concentrations for each trimester of pregnancy	Cross-lagged panels to examine the lagged and cross-sectional associations	(Ø) Stressful life events were not associated with hair cortisol concentrations
Harville et al. (2007)	100 pregnant individuals enrolled in a cohort study examining stress and physical activity and preterm birth (Pregnancy, Infection, and Nutrition [PIN] Study).	31% 31–35 years, 27% 26–30 years, 23% 20–25 years, 15% > 35 years, 4% < 20 years; 78% White, 17% Black, 5% Other; 37% completed more than college; 47% reported income *>* 400% of federal poverty level; 83% married	**Pregnancy stress** Three questions about recent stressors and rated daily stress on a 4-point scale Assessed each evening of saliva collection	**Salivary cortisol** Waking, 30 min after waking, 11 am, 5 pm, and 9 pm over three consecutive weekdays in either the early second trimester or late second trimester Calculated cortisol awakening response and total daytime cortisol output	Pearson’s correlations, adjusting for gestational age and time of day	(Ø) No significant associations of recent or daily stressors with cortisol levels or daily patterns
Harville et al. (2009)	1587 pregnant individuals enrolled in a cohort study examining stress and physical activity and preterm birth (Pregnancy, Infection, and Nutrition [PIN] Study)	32% 25–29 years, 29% 30–35 years, 19% 20–24 years, 12% *>* 35 years, 8% *<* 20 years; 71% White, 20% Black, 9% Other; 27% completed college degree; 48% reported income *>* 400% of federal poverty level; 74% married	**Pregnancy stress** Life Experiences Survey; Perceived Stress Scale Assessed in mid pregnancy (median=19 weeks, range = 18–20 weeks) and late pregnancy (median=27 and 28 weeks, range = 26–29 weeks)	**Salivary cortisol** Saliva samples collected between 8 am and 10 am at clinic visits in mid pregnancy (median=17 weeks, range = 16–18 weeks) and late pregnancy (median=26 weeks, range = 25–28 weeks)	Spearman correlations and hierarchical linear models adjusting for age, income as percent of the poverty line given reported household size, education, race, parity, marital status, smoking, pre-pregnancy BMI, pregnancy complications, and gestational age	(Ø) Neither stress measure was associated with cortisol
Hoffman et al. (2016)	90 pregnant individuals recruited to a prospective cohort study	Term births (n = 79) M age = 28.2 years (SD =6.1 years); 38% non-Hispanic White, 37% Hispanic White, 11% Native American 8% African American, 6% Other; modal educational attainment high school diploma/GED (44%); 59% married Preterm births (n = 11) M age = 32.4 years (SD = 5.8 years); 36% non-Hispanic White, 36% African American, 18% Hispanic White, 9% Native American; modal educational attainment high school diploma/GED (72%); 36% married	**Pregnancy stress** Perceived Stress Scale 14-item version Assessed at 16, 22, 28, 34, and 40 weeks gestation	**Hair cortisol concentrations** Hair samples collected three times in pregnancy at 16–18 weeks, 28–30 weeks, and 38–42 weeks to that reflected the hair cortisol concentrations the past three months	Regression models adjusting for race/ethnicity and tobacco use	(+) Perceived stress at 16 weeks gestation was associated with higher late second trimester hair cortisol (Ø) Perceived stress and hair cortisol concentrations at other timepoints
Kalra et al. (2007)	25 pregnant individuals recruited to a prospective cross-sectional pilot study in Toronto, Canada	Age range 18–45 years. Other sociodemographic characteristics were not reported	**Pregnancy stress** Perceived Stress Scale 10-item version Assessed in mid pregnancy (either end of the first or beginning of the second trimester)	**Hair cortisol concentrations** Hair samples collected at the end of the first or beginning of the second trimester reflecting the previous 1–1.5 months of pregnancy	Spearman’s rank order correlations	(+) Perceived stress was associated with significantly higher hair cortisol concentrations
King et al. (2022)	90 pregnant individuals in a longitudinal observational study of prenatal stress and child development (Brain and Behavior Infant Experiences [BABIES] project)	M age = 32.7 years (SD = 4.9 years, range = 21.0–44.4 years); 58.9% White, 20.0% Asian/Asian American, 8.9% Other Race, 3.3% Black/African American; 8.9% Hispanic/Latinx Ethnicity; 34.4% reported income > $150,000	**Pregnancy stress** Crisis in Family Systems – Revised Assessed between 12 and 37 weeks gestation, 3–8 weeks postpartum, and 5–8 months postpartum	**Hair cortisol concentrations** Hair samples collected at 12–37 weeks gestation, 3–8 weeks postpartum, and 5–8 months postpartum that reflected hair cortisol concentrations the past 5 months	Linear mixed effects model adjusting for hair chemical exposure/heat treatment, average frequency of hair washing, age, and season when hair was collected	(+) Within-person fluctuations in stress were associated with variation in hair cortisol concentrations across pregnancy and postpartum such that when stress was higher relative to an individual’s average, hair cortisol concentrations were also higher (Ø) Between-person fluctuations in stress were not associated with variation in hair cortisol
Kramer et al. (2009)	117 pregnant individuals enrolled in a larger multicenter prospective cohort study in the Montreal, Canada area	Sociodemographic characteristics of participants providing hair samples not reported.	**Proximal preconception and pregnancy stress** Crowding; Daily Hassles Scale; Marital Strain Scale; Abuse Assessment Screen; job-related stress; Perceived Stress Scale 10-item version Assessed in the second trimester (range = 24–26 weeks)	**Hair cortisol concentrations** Hair samples collected postpartum that reflected hair cortisol concentrations in the first trimester, second trimester, and third trimester Categorized as: (1) above versus at or below sample median; (2) by quartile	Multiple logistic regression adjusting for age, parity, cohabitation status, birthplace, cigarette smoking, language spoken at home, maternal education, family income, maternal height, pre-pregnancy BMI, and medical/obstetric risk	(Ø) None of the stress measures were associated with hair cortisol
la Marca-Ghaemmaghami et al. (2021)	51 pregnant individuals recruited to participate in a prospective longitudinal study in Zurich, Switzerland prior to 8 weeks gestation	M age = 30.0 years (range = 24.0–44.0); 56.9% completed university degree; 58.8% married, 98.0% cohabitating	**Pregnancy stress** 12-item Chronic Stress Screening Scale of the Trier Inventory for the Assessment of Chronic Stress Assessed in the first trimester (median=6.0 weeks, range = 4.0–9.0 weeks)	**Salivary cortisol** Waking, 30 min after waking, and 60 min after waking in the first trimester (78%) or second trimester (22%) Calculated cortisol awakening response	Spearman’s correlations adjusting for level of employment (0–25%, 25–50%, 50–75%, 75–100%, >100%)	(Ø) Chronic stress was not associated with morning levels of cortisol or the cortisol awakening response
Lazarides et al. (2020)	152 pregnant individuals were recruited for a prospective, longitudinal EMA-based study	M age = 27.8 years (SD = 5.5 years); 38.8% Non-Hispanic White, 38.1% Hispanic, 23.1% Other; 37.8% completed some college or associates degree; 36.6% income $50,000–100,000	**Pregnancy stress** Momentary stress assessed with Perceived Stress Scale EMA version; daily and monthly perceived stress assessed with the Perceived Stress 10-item version Assessed in early pregnancy (M=12.3 weeks, SD = 1.7 weeks), mid pregnancy (M= 19.3 weeks, SD = 1.3 weeks), and late pregnancy (M=28.7 weeks, SD = 1.3 weeks)	**Salivary cortisol** Waking, 30 min after waking, 12 pm, 4 pm, and 8 pm over the course of four days in early pregnancy (M=12.3 weeks, SD = 1.7 weeks), mid pregnancy (M=19.3 weeks, SD = 1.3 weeks), and late pregnancy (M=28.7 weeks, SD = 1.3 weeks)	Linear mixed models adjusting for time since waking, meal intake, weekend/weekday, pre-pregnancy BMI, and weeks gestation	(+) At the within-person level, momentary stress was positively associated with momentary cortisol. Cortisol output was greater on days when the subject reported higher day-level stress than their average stress level (Ø) No significant associations of stress with cortisol at the between-persons level
Musana et al. (2020)	133 pregnant individuals enrolled in a cross-sectional cohort study in Kenya	M age = 25.0 years, SD = 5.0 years; 64% married	**Pregnancy stress** Perceived Stress Scale 10-item Assessed in mid pregnancy (M=26 weeks, SD = 3 weeks)	**Hair cortisol concentrations** Hair samples collected in mid pregnancy (M=26 weeks, SD = 3 weeks) that reflected the prior three months of pregnancy	Hierarchical linear regression models adjusting for prenatal medical risk	(Ø) Perceived stress was not associated with hair cortisol
Obel et al. (2005)	650 Danish-speaking pregnant individuals recruited to a study of stressful life events in pregnancy and risk of preterm birth and intra-uterine growth restriction	44.5% 25–29 years, 36.2% > 29 years, 12.2% 20–24 years	**Pregnancy stress** Life Events Inventory; pregnancy-related stress. Dichotomized into individuals who experienced more than one life event, felt severely stressed by more than one life event, and women who experienced any pregnancy complications. Assessed in early pregnancy (median=14 weeks) and late pregnancy (median=30 weeks)	**Salivary cortisol** Morning and evening saliva samples in early pregnancy (median=14 weeks) and late pregnancy (median=30 weeks)	Independent samples t-tests and multiple linear regression, adjusting for gestational age, sample time, and smoking	(+) Individuals who either were very stressed by more than one life event in late pregnancy had significantly higher evening cortisol in late pregnancy. (+) Individuals who were worried about pregnancy in early pregnancy had significantly higher evening cortisol in early pregnancy. (Ø) Individuals who experienced more than one life event in pregnancy did not differ in evening cortisol levels. (Ø) Pregnancy stress measures were not associated with morning cortisol levels.
Pluess et al. (2012)	60 pregnant individuals enrolled in a prospective longitudinal study on child development in Trier, Germany	M age = 30.4 years (SD = 4.8 years, range = 16–39); 38.3% completed less then high school; 47.5% monthly household income 1500–3000 Euro; 91.7% living with partner	**Proximal preconception and pregnancy stress** Life Experiences Survey measured life events in the previous 12 months Assessed at 10–20 weeks gestation	**Salivary cortisol** Waking, 30 min after waking, 45 min after waking, and 60 min after waking on two different days during 35th and 36th week of gestation Calculated total morning cortisol output	Multiple linear regression adjusting for planned pregnancy and neuroticism	(Ø) Negative life events did not predict morning cortisol levels
Scharlau et al. (2018)	62 pregnant individuals enrolled in a larger longitudinal birth cohort study conducted in Leipzig, Germany (LIFE CHILD BIRTH cohort; Leipzig Research Center for Civilization Diseases Child Study)	37% 25–29 years, 27% 30–34 years, 21% < 25 years, 15% 35–30 years; 92% German nationality, 8% Other; 48% high socioeconomic status, 41% middle socioeconomic status, 11% low socioeconomic status	**Pregnancy stress** Patient Health Questionnaire – Stress subscale to assess stress in the prior 4 weeks Assessed in the stress subscale in the second (24–26 weeks) and third trimester (34–36 weeks)	**Hair cortisol concentrations** Hair samples collected in the second (24–26 weeks) and third trimester (34–36 weeks) that reflected hair cortisol concentrations the past month	Multiple linear regression models adjusting for BMI, offspring sex, hair washing, and location of hair sample	(Ø) Neither stress or changes in stress were associated with hair cortisol or changes in hair cortisol
Schreier et al. (2015b)	732 pregnant individuals enrolled in a pregnancy cohort study in Mexico City	M age = 27.4 years (SD = 5.6 years); 29.4% completed high school or junior college	**Proximal preconception and pregnancy stress** Crisis in Family Systems Questionnaire (past 6 months) Gestational timing of assessment not specified **Environmental pollution in pregnancy** Mercury exposure based on toenail clippings (evaluated as moderator) Measured during the second or third trimester	**Salivary cortisol** Waking, 45 min after waking, 10 h after waking, and at bedtime over two days in the second trimester (M=19.5 weeks, SD = 3.0 weeks) Calculated cortisol awakening response, slope, and total cortisol output	Multiple linear regression models to evaluate main and interactive effects adjusting for age, highest level of education, BMI, and gestational age	(⊗) Individuals above the median for mercury exposure (higher environmental pollution) and stress had a blunted morning cortisol response compared to women exposed to high stress but lower mercury levels (lower environmental pollution)
Simon et al. (2016)	30 participants enrolled in a supplementary pilot study to a longitudinal pregnancy cohort study (Stress in Pregnancy Study) designed to clarify the relationship between maternal stress and pregnancy outcomes	12 African American women (M age = 30.8 years, SD = 4.8 years; 75% completed college education; 83% household income >$30,000) 18 Caucasian women (M age = 29.9 years, SD = 6.6 years; 66.7% completed college education; 61.1% household income >$30,000)	**Proximal preconception and pregnancy stress** The Home Hardship Scales; the Stressful Life Events Scale; the USDA Food Security Scale; Krieger Perceived Discrimination Scale; Misra Stress Scale; Perceived Stress Scale Assessed in the third trimester	**Salivary cortisol** Waking, 30 min after waking, and bedtime over two days in the third trimester Calculated cortisol awakening response	Multivariate linear regression models adjusting for age, parity, income, and BMI	(−) Higher perceived stress was associated with a lower cortisol awakening response (Ø) Other measures of stress with cortisol levels and cortisol awakening response
Smew et al. (2018)	92 pregnant individuals enrolled in a larger prospective longitudinal birth cohort study (Born into Life) in Stockholm, Sweden	M age = 32.5 years (SD = 3.7 years); 88.5% completed university level education	**Pregnancy stress** Perceived Stress Scale 10-item version Assessed at 26–28 weeks gestation	**Salivary cortisol** Evening before a lab visit and the morning of the lab visit at 26–28 weeks gestation. Calculated decline from morning to evening	Pearson’s correlations	Higher perceived stress was associated with: (−) lower morning cortisol levels (−) a smaller cortisol decline from morning to evening (Ø) Perceived stress with evening cortisol levels
Suglia et al. (2010)	200 pregnant individuals enrolled in a prospective pregnancy cohort study (Asthma Coalition on Community, Environment, and Social Stress)	M age = 26.7 years (SD = 5.9 years); 66% Hispanic, 34% Black; 41.5% completed less than high school	**Proximal preconception and pregnancy stress** Cumulative stress calculated based on recent (past year) interpersonal violence, discrimination, negative life events, and exposure to violence. Participants were given a score of 1 if they were in the upper quartile for each measure. Summed to create a cumulative stress score Assessed in the second or third trimester	**Salivary cortisol** Waking, 30–90 min after waking, 3–6.5 h after waking, 7.5–11.5 h after waking, and 11.5 h after waking over three days in mid pregnancy (~25 weeks) Calculated cortisol awakening response, slope, and total cortisol output	Linear mixed models adjusting for age, education, smoking, pre-pregnancy BMI, and gestational age at saliva sampling	Higher cumulative stress was associated with: (−) lower morning cortisol levels* (+) a flatter diurnal slope* *Among Black participants, but not among Hispanic participants
Urizar et al. (2004)	41 pregnant individuals at an outpatient prenatal clinic recruited to receive a pilot stress reduction intervention	M age = 26.0 years (SD = 4.6 years); 81% Spanish-speaking Latina; 68% earned annual income less than $20,000; 71% married or living with a partner	**Pregnancy stress** Participants reported on stress on a 100-point analog scale (0: not at all stressed, 100: extremely stressed) Assessed on four days of a ten-day study period. Participants reported on stress 2 days before stress reduction intervention (day 1, day 2; baseline) and on 2 days after the stress reduction intervention (day 9, day 10). Stress reduction intervention occurred in late pregnancy (M=27 weeks, SD = 8.0)	**Salivary cortisol** 45 min after waking up and at 8 pm on four days of a ten-day study period Participants collected samples 2 days before stress reduction intervention (day 1, day 2) and on 2 days after the stress reduction intervention (day 9, day 10)	Pearson’s correlations adjusting for gestational age	(Ø) Pregnancy stress was not significantly associated with morning or evening cortisol levels
Urizar et al. (2019)	100 pregnant individuals enrolled in a randomized controlled trial of a prenatal cognitive behavioral stress management intervention	M age = 27 years, SD = 6.26, range = 18–40 years; 71% Latina, 18% African American, 4% Asian American, 4% Non-Hispanic White, 3% Mixed Ethnicity; 71% completed high school or less; 76% annual family combined income < $20,000. 51% single	**Pregnancy stress** Perceived Stress Scale 14-item version Assessed prior to the intervention (M=10.0 weeks, SD = 4.3 weeks, range = 2–17 weeks)	**Salivary cortisol** Waking, 30 min after waking, 45 min after waking, 60 min after waking, 12 pm, 4 pm, and 8 pm over one day in early pregnancy, the second trimester, and third trimester Calculated cortisol awakening response, slope, and total cortisol output	Pearson’s correlations, adjusting for ethnicity and pregnancy anxiety	(+) Higher perceived stress associated with a greater cortisol awakening response during the third trimester of pregnancy (Ø) Perceived stress was not associated with the cortisol awakening response at other gestational timepoints or other measures of diurnal cortisol
Valladares et al. (2009)	147 pregnant individuals in a larger ongoing demographic surveillance study conducted in an urban area of Nicaragua	Age range 14–40 years (33% adolescents); 71% “low socio-economic conditions”	**Pregnancy stress** Violence during pregnancy and perceived stress at in home interviews with trained field workers Assessed between 18 and 29 weeks gestation	**Salivary cortisol** Morning (7–8 am) and afternoon (2–3 pm) on one day at 18–39 weeks gestation	Structural equation modeling using unstandardized residuals of cortisol after adjustment for gestational age at saliva sampling	(+) Higher perceived stress was associated with an increase in the standardized residuals of afternoon cortisol levels (+) Violence during pregnancy was associated with higher afternoon cortisol levels (Ø) Perceived stress and violence during pregnancy were not associated with morning cortisol levels
Voegtline et al. (2013)	112 pregnant individuals recruited through advertisements posted in a local university and hospital	M age = 31.2 years (SD = 4.6 years); 80% Caucasian, 13% Black; 7% Asian; M education= 17.2 years (SD = 2.1 years); 91% married	**Pregnancy stress** Pregnancy Experiences Scale to measure pregnancy hassles Assessed at 5 prenatal visits spanning 24 weeks to 38 weeks gestation	**Salivary cortisol** Approximately 30 min after arrival to the laboratory between 2 pm and 4 pm in the afternoon at 5 prenatal visits spanning 24 weeks to 38 weeks gestation	Partial correlations adjusting for time of day	(Ø) Stress was not associated with salivary cortisol levels
**Studies including measures of both childhood stress and proximal preconception and pregnancy stress (*n* = 11)**						
Bowers et al. (2018)	30 pregnant individuals recruited to a longitudinal cohort study on the association between maternal life-course distress and offspring development in a sample with high sociodemographic risk (Pregnancy and Infant Development [PRIDE] Study)	M age = 21.6 years (SD = 3.5 years); 46.7% White, 43.3% Black, 10.0% Other; 6.7% Hispanic; 100% single	**Childhood stress** Adverse Childhood Experiences Questionnaire 10-item version Retrospective report in mid pregnancy **Pregnancy stress** Pregnancy Experiences Scale Brief version; Perceived Stress Scale Assessed in mid pregnancy (gestational weeks not specified)	**Hair cortisol concentrations** Hair sample in mid pregnancy (gestational weeks not specified) reflecting the prior three months of pregnancy	Linear regression models, adjusting for age and race	(−) Adverse childhood experiences were inversely associated with hair cortisol (⊗) Perceived stress associated with hair cortisol concentrations among individuals who experienced 2 or more adverse childhood experiences but not those who experienced fewer
Bublitz and Stroud (2013)	41 pregnant individuals who were enrolled in a larger study of the effects of maternal mood on fetal and infant development (Behavior and Mood in Mothers, Behavior in Infants [BAMBI] Study) recruited to a pilot study on the links between daily stress and maternal cortisol	M age = 26 years (SD = 5 years); 63% Caucasian; 45% total household annual income < $30,000; 69% less than a college degree; 55% unmarried	**Childhood stress** Adverse Childhood Experiences Questionnaire 15-item version Retrospective report during pregnancy **Pregnancy stress** Pregnancy Experiences Scale Assessed each day for three days after three study sessions at 20 weeks (SD = 1 week), 28 weeks (SD = 1 week), and 35 weeks (SD = 1 week) gestation	**Salivary cortisol** Waking, 30 min after waking, and in the evening over three consecutive days after each study visit at 20 weeks (SD = 1 week), 28 weeks (SD = 1 week), and 35 weeks (SD = 1 week) gestation. Calculated cortisol awakening response	Hierarchical linear models adjusting for time of cortisol sampling and gestational age	(⊗) Prior day stress was associated with increased cortisol 30 min after waking cortisol in women with childhood sexual abuse histories compared to women with no history of childhood abuse (Ø) Child abuse history did not moderate the association between pregnancy stress and waking, cortisol awakening response, or evening cortisol
Bublitz et al. (2016)	17 pregnant individuals recruited from a larger study of maternal smoking and infant behavior (Behavior and Mood in Babies and Mothers II) and enrolled in a pilot study on the impact of everyday stressful experiences on gestational length	6 participants reported a childhood maltreatment history and 11 participants reported no history of childhood maltreatment. M age = 25 years (SD = 3 years); 27% White, 19% Hispanic, 18% Black, 12% American Indian/Alaska Native, 24% Other; 41% annual income less than or equal to $19,999; 33% married	**Childhood stresss** Adverse Childhood Experiences Questionnaire. Endorsing physical/sexual abuse qualified as having a maltreatment history Retrospective report during pregnancy **Pregnancy stress** Momentary stress level recorded via text message at semi-random intervals Assessed four times a day between 9 am and 6 pm for two days following a study session in mid pregnancy (M=27 weeks, SD = 2 weeks) and late pregnancy (M=34 weeks, SD = 1 week) 16 total stress assessments per participant	**Salivary cortisol** 30 min following each measure of momentary stress (16 times/participant)	Hierarchical linear models adjusting for parity	(⊗) As momentary stress increased, cortisol concentrations decreased for women with maltreatment history whereas for women without maltreatment histories, as momentary stress increased, cortisol concentrations increased
Epstein et al. (2020)	58 pregnant individuals recruited to a cross-sectional study from an urban hospital-based obstetric clinic	M age = 27.9 years (SD = 5.3 years); 60% Caucasian, 22% Black or African American, 7% Asian, 10% Other; 9% Hispanic Ethnicity; 52% completed college; 48% household income < $40,000; 50% married	**Childhood and adulthood stress** Cumulative exposure to stress over the life course with the Stress and Adversity Inventory categorized by timing (childhood vs. adulthood) Assessed in mid pregnancy (M=26.5 weeks, range = 20–28 weeks)	**Salivary cortisol** Waking, 30 min after waking, before lunch, before dinner, and before bedtime over three consecutive days at an average of 26.5 weeks gestation Calculated cortisol awaking response, slope, and total cortisol output	Multiple linear regression models to evaluate main effects and interactive effects of childhood stress and adulthood stress on cortisol levels adjusting for age and smoking	(+) More childhood stressors were associated with a greater cortisol awakening response. (⊗) More adulthood stressors were associated with a steeper cortisol slope and blunted cortisol awakening response only among individuals with high childhood stress. (Ø) Neither childhood nor adulthood stressors were associated with total cortisol output. (Ø) Interactive effect of childhood and adulthood stress on total cortisol output.
Jairaj et al. (2020)	118 participants enrolled in a prospective longitudinal case-control study examining HPA axis output in pregnant women and postpartum in Dublin	Individuals with major depression in pregnancy (M age = 31.6 years, SD = 3.8 years; 95.7% Caucasian; 68.2% completed undergraduate education; 47.8% married); Individuals with a history of depression who were not depressed in pregnancy (M age = 32.4 years, SD = 5.2 years; 94.1% Caucasian; 51.5% completed undergraduate education; 69.7% married); Nondepressed control (M age = 33.9 years, SD = 4.7 years; 88.4% Caucasian; 55.8% completed undergraduate education; 7.9% single, 30.2% partnership, 62.8% married)^a^	**Childhood stress** Childhood Trauma Questionnaire Retrospective report at 20–30 weeks gestation **Pregnancy stress** Perceived Stress Scale Assessed at 20–30 weeks gestation.	**Salivary cortisol** Waking, 30 min after waking, 45 min after waking, 12 h after waking, and 12.5 h after waking over one day between 20 and 30 weeks gestation (M=26 weeks) Calculated area under the curve with respect to ground and area under the curve with respect to increase	Spearman rankorder correlation adjusting for depressive symptoms	(+) Childhood trauma associated with higher evening cortisol levels. (Ø) Childhood trauma was not associated with other measures of tonic cortisol or area under the curve measures.
King et al. (2008)	214 pregnant individuals recruited from the community and selected for trauma exposure and lifetime PTSD diagnosis	M age = 32.7 years (range = 21.0–44.4); 53% White, 18% Asian American, 3% Black/African American, 2% Native Hawaiian/Pacific Islander, 8% Another race, 8% Hispanic/Latinx Ethnicity; modal annual income > $150,000 (31%)	**Childhood stress** Life Stressor Checklist **Proximal preconception and pregnancy stress** Abuse Assessment Screen to assess past year intimate partner violence Gestational timing of assessment not specified.	**Salivary cortisol** Evening prior to eating dinner (4–6 pm) Timing of assessment not specified	Multivariate linear regression models adjusting for endocrine disorders, taking steroid or antidepressant medication, steroid hormones that vary by gestational age, smoker status	(Ø) Childhood abuse history and past year domestic violence were not significantly associated with evening cortisol levels
Orta et al. (2019)	97 pregnant individuals drawn from a prospective cohort study consisting of individuals attending prenatal clinics in Lima, Peru recruited in early pregnancy (Pregnancy Outcomes, Maternal, and Infant Study [PrOMIS])	M age = 26.5 years (SD = 5.8 years); 85.6% Mestizo ethnicity; 79.4% married or living with a partner	**Childhood stress** Item assessing history of physical or sexual abuse prior to age 18 (yes/no) Retrospective report in first trimester **Pregnancy stress** Items assessing difficulty accessing basic goods (yes/no), unemployment (yes/no), educational attainment (12 years/>12 years), lifetime experiences of intimate partner violence (yes/no), and perceived stress with the Perceived Stress Scale 14-item version Assessed in the first trimester	**Hair cortisol concentrations** Hair samples collected at delivery and segmented to reflect hair cortisol concentrations in the first trimester, second trimester, and third trimester	Multivariate linear regression models adjusting for early pregnancy BMI, alcohol consumption, hair structure, hair treatment, and UV exposure Sensitivity analysis adjusted for gestational age	(Ø) None of the stress measures were associated with hair cortisol
Shea et al. (2007)	66 pregnant individuals enrolled in an ongoing clinical cohort study in Canada (Maternal Adversity, Vulnerability, and Neurodevelopment [MAVAN]) that included participants presenting symptoms of depression and control participants	33 non-depressed control participants M age = 31.9 years (SD = 4.3 years); 100% married 14 depressed participants M age = 29.8 years (SD = 4.8 years); 93% married 19 depressed/anxious participants M age = 28.6 years (SD = 6.1 years); 100% married	**Childhood stress** Childhood Trauma Questionnaire Retrospective report in mid pregnancy (M=20 weeks, range = 12–24 weeks) **Proximal preconception and pregnancy stress** Inventory for Recent Life Stressful Events (past 6 months) Assessed in mid pregnancy (M=20 weeks, range = 12–24 weeks)	**Salivary cortisol** Waking, 30 min after waking, and 60 min after waking over two days in mid to late pregnancy (M=28.4 weeks, range = 25–33 weeks) Calculated cortisol awakening response	Linear regression models adjusting for wake time and antidepressant use	(−) Childhood trauma was associated with lower cortisol levels at waking (Ø) Childhood trauma was not associated with the cortisol awakening response
Stephens et al. (2021)	178 pregnant individuals enrolled in a larger prospective longitudinal study collecting measures of stress and health (Measurement of Maternal Stress [MOMS] Study)	M age = 30.1 years (SD = 5.1; range = 19.7–44.7); 80.9% White, 16.3% Black, 2.8% Other; Income 12.6% < $15,000; 26.4% $15,000–49,999; 37.9% $50,000–99,999, 23.0% ≥ $100,000; 83.7% married	**Childhood stress** Childhood Trauma Questionnaire; childhood economic disadvantage Retrospective report in mid pregnancy (range = 12–21 weeks) **Proximal preconception and pregnancy stress** Stressful Life Events Schedule to assess life events in the past year; current economic disadvantage Assessed in mid pregnancy (range = 12–21 weeks)	**Salivary cortisol** Waking, 30 min after waking, 12 pm, 4 pm, 8 pm, and at bedtime over four days in mid pregnancy (range = 12–21 weeks) Calculated cortisol awakening response and slope	Hierarchical linear models adjusting for smoking status, relationship status, gestational age, maternal age, BMI, parity, and race/ethnicity that included interactive effect between childhood and proximal preconception/pregnancy stress	(+) Moderate/severe childhood maltreatment, specifically childhood sexual abuse, was associated with a greater cortisol awakening response Childhood economic disadvantage, not maltreatment, was associated with: (+) a flatter cortisol slope (−) less flattening of the cortisol slope across pregnancy (Ø) Interactive effect between childhood and proximal preconception/pregnancy stress
Swales et al. (2018)	90 pregnant individuals recruited from a large university medical center where they had been admitted for risk of imminent preterm birth	M age = 30.0 years (SD = 6.4 years); 68.9% Hispanic, 21.1% Non-Hispanic White; modal income $0-$30,000 (37.8%); 76.7% cohabitating with a partner	**Childhood, adulthood, and pregnancy stress** Lifetime life events with the Life Events Checklist Four sum scores were calculated based on the number of endorsed life events in each age group of childhood, adolescence, adulthood, and the current pregnancy Retrospective report in mid to late pregnancy (M=28.6 weeks, SD = 3.20 weeks)	**Hair cortisol concentrations** Hair sample in mid to late pregnancy that reflected hair cortisol concentrations in the prior three months of pregnancy (M=28.6 weeks, SD = 3.20 weeks)	Linear regression models testing main and interactive effects of traumatic events across development, adjusting for gestational age	(+) Traumatic life events in childhood and adulthood independently predicted higher hair cortisol concentrations. (⊗) Adult exposure to traumatic life events was more strongly positively associated with hair cortisol among individuals who experienced one or more traumatic events during childhood. (Ø) Traumatic events in adolescence and pregnancy did not predict hair cortisol.
Thomas et al. (2018b)	356 pregnant individuals enrolled in a prospective pregnancy cohort (Alberta Pregnancy Outcomes and Nutrition [APrON])	M age = 31.8 years (range = 21–43); 84.9% White Caucasian, 3.7% Latin American, 3.1% Chinese, 8.3% Other; modal household income ‘more than $100,000/year’ (57.8%); 88.5% married	**Childhood stress** Adverse Childhood Experiences Questionnaire 10-item version Retrospective report during pregnancy **Pregnancy stress** Stressful Life Events Questionnaire Stressful life events assessed at each pregnancy visit	**Salivary cortisol** Waking, 30 min after waking, midday, and bedtime over several days in the first trimester (M=11.4 weeks), second trimester (M=17.1 weeks), and third trimester (M=32.4 weeks) Calculated cortisol awakening response, daytime slope, and total daytime cortisol output	Multilevel models adjusting for socioeconomic status, parity, and maternal age Secondary analyses (1) included stressful life events in pregnancy and maternal psychological distress in pregnancy as additional covariates and (2) examined the interaction between adverse childhood experiences and stressful life events in pregnancy	Adverse childhood experiences were associated with: (+) increased cortisol levels 30 min after waking (+) a flatter slope* (+) increased cortisol awakening response in early pregnancy (−) less flattening of the diurnal slope across pregnancy* (Ø) Interactive effect between childhood and pregnancy stress (Ø) Adverse childhood experiences were not associated with waking cortisol levels, the cortisol awakening response in late pregnancy, changes in the cortisol awakening response, total cortisol output, and changes in cortisol output
**Studies including measures of lifetime stress (*n* = 4)**						
Bosquet Enlow et al. (2017)	195 pregnant individuals enrolled in prospective pregnancy cohort designed to examine the role of prenatal stress on stress responses and respiratory health in children (Programming of Intergenerational Stress Mechanisms [PRISM])	M age = 31.21 years (SD = 5.51 years); 34% White, 25% Black/Haitian, 34% Hispanic, 7% Other; 31% obtained high school diploma	**Lifetime stress** 30-item Life Stressor Checklist-Revised **Pregnancy stress** Crisis in Family Systems-Revised survey Both assessed within two weeks of study enrollment (M study enrollment = 26.9 weeks, SD = 8.1 weeks)	**Hair cortisol concentrations** Hair samples within one week of delivery that reflected hair cortisol concentrations in the third trimester	Spearman correlation coefficients	(Ø) Lifetime stressful events were not associated with hair cortisol concentrations
Campbell et al. (2019)	455 participants enrolled in a pregnancy cohort designed to examine effects of prenatal chemical and non-chemical exposures on childhood asthma risk (Asthma Coalition on Community, Environment and Social Stress [ACCESS] project)	M age = 27.5 years (SD = 5.8 years); 60% Hispanic, 28% Black, 9% White, 4% other or multiple races; 65.9% completed high school	**Lifetime stress** Revised Conflict Tactics Scale short form. Any affirmative answer considered as having a history of trauma. Divided into development stage: childhood, adolescence, adulthood before pregnancy, pregnancy. Retrospectively reported during pregnancy.	**Salivary cortisol** Waking, 45 min after waking, 4 h after waking, 10 weeks after waking, and at bedtime over three consecutive week days in late pregnancy (M=29.0 weeks, SD = 5.1 weeks). Calculated cortisol awakening response and diurnal slope	Linear regression models adjusting for maternal age, race, education, pre-pregnancy BMI, and gestational age	(+) History of lifetime trauma associated with higher afternoon cortisol levels. No differences by developmental stage (Ø) History of lifetime trauma with other measures of tonic cortisol, cortisol awakening response, and diurnal slope
Ghosn et al. (2019)	101 pregnant individuals enrolled in a prospective cohort study	Forty-two participants had a trauma history (M age = 31.9 years, SD = 5.2 years) and 59 did not have a trauma history (M age = 31.6 years, SD = 4.5 years)	**Lifetime stress** The Trauma Questionnaire assessed history of 19 DSM Criterion A stressors Assessed at 38 weeks gestation.	**Salivary cortisol** Between 10 am and 12 pm. Collected at 38 weeks gestation	Quantile regression models tested differences in cortisol levels by trauma group adjusting for age and parity	(+) Individuals with a trauma history had higher morning levels compared to the non-trauma group
Schreier et al. (2016)	180 pregnant individuals enrolled in prospective pregnancy cohort designed to examine the role of prenatal stress on stress responses and respiratory health in children (Programming of Intergenerational Stress Mechanisms [PRISM])	M age = 31.03 years (SD = 5.41 years); 45.6% Hispanic, 35.6% White, 18.9% Black; 25% completed less than high school, 24.4% completed graduate education, 21.1% completed undergraduate, 18.9% completed some college, 10% completed high school	**Lifetime stress** 30-item Life Stressor Checklist Calculated two scores: score of all endorsed lifetime stressful events and a score of lifetime traumatic events Assessed within 2 weeks of study enrollment (M=26.9 weeks, 8.1 weeks).	**Hair cortisol concentrations** Hair samples collected after delivery segmented to reflect hair cortisol concentrations in all three trimesters (55.6%), in the second and third trimester (36.1%), and in the third trimester only (8.3%)	Mixed effects models adjusting for inhaled corticosteroid use, pre-pregnancy BMI, PTSD and depressive symptoms, and race/ethnicity Secondary analyses stratified by race/ethnicity	(+) Lifetime traumatic events were associated with higher maternal hair cortisol. In stratified analyses, association was only significant in Black women but not White or Hispanic women (Ø) Lifetime stressful events were not associated with hair cortisol levels

Note. Studies took place in the United States unless otherwise noted. Sociodemographic characteristics presented were based on author report in original manuscript and therefore vary across studies. 11 of the 48 reports included samples from prospective, longitudinal cohort studies (n = 3 APRON cohort; n = 3 PRISM cohort; n = 3 BAMBI cohort; n = 2 PiN cohort).

BMI = body mass index

a Authors only report sociodemographic characteristics by subgroup; analyses conducted in full sample.

**Table 4:** Limitations of existing literature and recommendations for future research.

Limitation	Recommendation (s)

Variation in operationalization of stress	Further precision in conceptualization and measurement of stress Evaluate potential differences by type of stress and synergistic effects of different types of stress across developmental periods
Measurements of cortisol limited to one day, only one sample per day, or one gestational assessment	Repeated sampling of cortisol across multiple days and gestational timepoints to increase precision and account for changes in cortisol indices across pregnancy
Measurements of stress limited to one gestational assessment	Repeated measures of stress across gestation to account for changes in stress and/or perceptions of stress across pregnancy that may show distinct links with cortisol
Variation in covariates included (if any) in analytic models	Statistically controlling for confounding factors with demonstrated links to cortisol in pregnancy
Focus on between-person associations of stress with cortisol levels	Employ person-centered approaches or multilevel modeling to parse within- and between-person associations of stress and cortisol and account for individual differences

## Footnotes

1 The demographic characteristics of each sample presented in the current review are based on the information authors provided thus vary across studies.

2 Although all participants were a part of the APRON cohort, the sample size and inclusion criteria for analysis across studies varied. Thomas-Arygriou et al. (2021) included participants who were less than 27 weeks gestation whereas the other two reports included participants who were less than 22 weeks gestation.

3 Daily and monthly cortisol levels were operationalized with area under the curve measures of total cortisol output.

4 Bosquet et al. (2017) (n = 195) included individuals who identified as Black, White, Hispanic, or multiracial whereas Schreier et al. (2016) (n = 180) excluded individuals who identified as multiracial to conduct subgroup analyses by race/ethnicity.
